# The role of cortisol in ischemic heart disease, ischemic stroke, type 2 diabetes, and cardiovascular disease risk factors: a bi-directional Mendelian randomization study

**DOI:** 10.1186/s12916-020-01831-3

**Published:** 2020-11-27

**Authors:** Man Ki Kwok, Ichiro Kawachi, David Rehkopf, Catherine Mary Schooling

**Affiliations:** 1 School of Public Health, Li Ka Shing Faculty of Medicine, The University of Hong Kong, 1/F, Patrick Manson Building (North Wing), 7 Sassoon Road, Hong Kong Special Administrative Region, China; 2 Department of Social and Behavioral Sciences, Harvard T. H. Chan School of Public Health, Boston, MA USA; 3 Department of Medicine, Stanford University, Stanford, CA USA; 4 City University of New York Graduate School of Public Health and Health Policy, New York, USA

**Keywords:** Cortisol, Cardiovascular disease, Diabetes, Risk factors, Mendelian randomization

## Abstract

**Background:**

Cortisol, a steroid hormone frequently used as a biomarker of stress, is associated with cardiovascular disease (CVD) and type 2 diabetes mellitus (T2DM). To clarify whether cortisol causes these outcomes, we assessed the role of cortisol in ischemic heart disease (IHD), ischemic stroke, T2DM, and CVD risk factors using a bi-directional Mendelian randomization (MR) study.

**Methods:**

Single nucleotide polymorphisms (SNPs) strongly (*P* < 5 × 10^−6^) and independently (*r*^2^ < 0.001) predicting cortisol were obtained from the CORtisol NETwork (CORNET) consortium (*n* = 12,597) and two metabolomics genome-wide association studies (GWAS) (*n* = 7824 and *n* = 2049). They were applied to GWAS of the primary outcomes (IHD, ischemic stroke and T2DM) and secondary outcomes (adiposity, glycemic traits, blood pressure and lipids) to obtain estimates using inverse variance weighting, with weighted median, MR-Egger, and MR-PRESSO as sensitivity analyses. Conversely, SNPs predicting IHD, ischemic stroke, and T2DM were applied to the cortisol GWAS.

**Results:**

Genetically predicted cortisol (based on 6 SNPs from CORNET; *F*-statistic = 28.3) was not associated with IHD (odds ratio (OR) 0.98 per 1 unit increase in log-transformed cortisol, 95% confidence interval (CI) 0.93–1.03), ischemic stroke (0.99, 95% CI 0.91–1.08), T2DM (1.00, 95% CI 0.96–1.04), or CVD risk factors. Genetically predicted IHD, ischemic stroke, and T2DM were not associated with cortisol.

**Conclusions:**

Contrary to observational studies, genetically predicted cortisol was unrelated to IHD, ischemic stroke, T2DM, or CVD risk factors, or vice versa. Our MR results find no evidence that cortisol plays a role in cardiovascular risk, casting doubts on the cortisol-related pathway, although replication is warranted.

**Supplementary information:**

**Supplementary information** accompanies this paper at 10.1186/s12916-020-01831-3.

## Background

Stress is commonly known as a risk factor for cardiovascular disease (CVD) [1], but its causal role has been questioned [2]. Assessing stress hormones provides an alternative approach to self-report [3]. In response to stress, cortisol, the major stress hormone, is elevated through activation of the hypothalamic-pituitary-adrenal (HPA) axis [4], as demonstrated by a review of psychological experiments showing that uncontrollable and potentially negatively judged tasks raise cortisol [5]. Elevated cortisol elicits extensive physiological responses, including energy mobilization (via promoting blood glucose followed by breakdown of proteins and fat) and homeostasis maintenance (via inducing vasoconstriction and sodium retention) [6]. It initially results in a loss of appetite and weight loss but, if prolonged, may promote over-eating and weight gain [7]. People with Cushing’s syndrome who are chronically exposed to excess glucocorticoids, primarily due to medication, tend to have more abdominal fat and weight gain [8]. Thus, there are highly plausible biological pathways through which stress, through elevated cortisol, may impact CVD.

Observationally, higher plasma cortisol to testosterone ratio has been found associated with ischemic heart disease (IHD) incidence and mortality, but the association could be mediated by CVD risk factors [9]. Hair cortisol has also been positively associated with cardiovascular medication usage and type 2 diabetes mellitus (T2DM) [10]. People with dyslipidemia, hypertension, or hyperglycemia had higher urinary cortisol metabolites, but not plasma cortisol [11]. Receiving glucocorticoids or developing Cushing’s syndrome was associated with incident IHD [12]. Receiving glucocorticoids among people with Addison’s disease (cortisol insufficiency) was also associated with higher HbA1c and a poor lipid profile, but not abdominal fat [13].

However, whether cortisol is an actual cause of CVD is unknown, considering observational studies are inherently open to confounding and reverse causality and can be open to selection bias. To date, two large observational studies have shown glucocorticoid prescription associated with higher CVD risk (OR ranging from 1.22 to 2.56) [14, 15], but no adequately large randomized controlled trials (RCTs) have examined the effect of glucocorticoids on incident CVD.

When definitive evidence is lacking, Mendelian randomization (MR) provides an alternative approach from observational studies by taking advantage of randomly assigned genetic variation at conception as a proxy of exposure preceding onset of disease [16]. Recently, an MR study found genetically predicted subjective well-being did not affect IHD or CVD risk factors [17], consistent with a Bayesian network study that pruned indirect effects which found depression may not directly affect T2DM or other CVD risk factors [18]. Particularly for cortisol, a recent MR study suggested genetically predicted cortisol was positively associated with IHD, but its two-sample MR estimates based on limited genetic instruments (*n* = 3) and its one-sample MR estimates based on three small European cohorts had 95% confidence interval (CI) including the null [19]. Another MR showed genetically predicted cortisol based on two genetic instruments positively associated with IHD using one-sample MR among healthy participants and patients with suspected or confirmed IHD [20]. However, no MR study has explicitly considered the effect of cortisol on IHD using more comprehensive genetic predictors of cortisol, the effect on ischemic stroke, T2DM, and other CVD risk factors or whether cortisol may be a symptom rather than consequence of IHD, ischemic stroke, and T2DM. Here, we assessed the role of cortisol in cardiovascular disease risk using bi-directional two-sample MR, i.e., we assessed whether genetically higher cortisol was associated with IHD, ischemic stroke, T2DM, and other CVD risk factors; conversely, we assessed whether genetically higher risk of IHD, ischemic stroke, and T2DM was associated with cortisol.

## Methods

### Association of genetically predicted cortisol with IHD, ischemic stroke, T2DM, and other CVD risk factors

#### Genetically predicted cortisol

Single nucleotide polymorphisms (SNPs) strongly (*P* < 5 × 10^−6^) and independently (*r*^2^ < 0.001) associated with morning fasting plasma/serum cortisol were used as genetic instrumental variables. Independent variants (*r*^2^ < 0.001) were selected using the “clump_data” function of the MR-Base R package. Non-bialleleic or indel genetic variants or those without a rs number were excluded. The genetic variants were obtained from three genome-wide association studies (GWAS) of people of European descent considered separately because the unit for cortisol varied between GWAS. The three GWAS were (1) the CORtisol NETwork (CORNET) consortium of 12,597 participants from 11 Western European population-based cohorts (mean age 53.5 years, 59.2% women), with *z*-score (standard deviation (SD) score) of log-transformed plasma cortisol adjusted for age and sex and corrected for genomic control [21], with genetic association with cortisol estimates download from https://datashare.is.ed.ac.uk/handle/10283/2787 as in Crawford et al. [19]; (2) a metabolomics GWAS by Shin et al. of 7824 participants (*n* = 7795 with cortisol tested) from the TwinsUK and a Southern German study (mean age 55.1 years, 83.5% women) with log-transformed plasma/serum cortisol adjusted for age, sex, and/or batch and corrected for genomic control [22]; and (3) a metabolomics GWAS by Long et al. of 2049 participants from the TwinsUK (one third of the sample was included in the Shin GWAS but with newer genotyping array and metabolomics profiling technologies) (median age 58.0 years, 96.6% women) with log-transformed mean of median normalized serum cortisol from three visits adjusted for age and sex [23]. All three GWAS including CORNET, Shin, and Long GWAS measured morning cortisol from blood samples collected at a comparable time of the day after fasting. Given CORNET is the largest GWAS of cortisol, we used SNPs from CORNET for the main analysis and genetic predictors from the Shin and Long for replication. In addition, we used three alternative approaches as secondary analyses. Firstly, based on the originally identified strong and independent SNPs for cortisol from each of the three cortisol GWAS, we used estimates derived from *P* value based effect sizes and correction for sample overlap [24]. Secondly, we used estimates obtained from genetic associations with cortisol from Crawford et al. [19]. Thirdly, we conducted a meta-analysis of all SNPs available in the three cortisol GWAS with a conversion of all estimates from different sources into the same unit using the *P* value based effect sizes with correction for sample overlap, and then identified SNPs which were strongly and independently associated with cortisol from this meta-analysis [25]. Proxy SNPs (*r*^2^ ≥ 0.8) in Europeans obtained from LDLink [26] were used for any SNP unavailable for an outcome. Palindromic SNPs coded A/T or C/G were aligned on effect allele frequency. To address possible known horizontal pleiotropy, any associations of the cortisol SNPs with CVD risk factors (*P* < 5 × 10^−8^ and *r*^2^ ≥ 0.8) were identified using PhenoScanner [27], and these SNPs were excluded in a sensitivity analysis.

#### Genetic associations with IHD, ischemic stroke, T2DM, and CVD risk factors

The primary outcomes were IHD, ischemic stroke, and T2DM. Genetic associations with IHD in people of European descent were obtained from CARDIoGRAMplusC4D 1000 Genomes-based GWAS (cases = 60,801, controls = 123,504) primarily of European descent (77%), followed by South Asian (13%), East Asian (6%), and Hispanic/African American descent (~ 4%), adjusted for genomic control [28]. We then replicated the analysis using the UK Biobank GWAS of IHD (cases = 31,355, controls = 377,103), adjusted for birth year, sex, and four principal components [29]. The UK Biobank recruited 503,317 adults (94% European ancestry) intended to be aged 40 to 69 years between 2006 and 2010 [30]. Genetic associations with ischemic stroke in Europeans were obtained from MEGASTROKE (cases = 40,585, controls = 406,111) (mean age 67.4 years, 41.7% women from the full trans-ethnic studies including Europeans), adjusted for age, sex, and study-specific covariates and corrected for genomic control [31]. The UK Biobank included in our analyses was from participants of British white descent [29]. The UK Biobank GWAS of ischemic stroke was not used for replication because of relatively few cases (*n* = 3314) [32]. Genetic associations with T2DM were obtained from DIAbetes Meta-ANalysis of Trans-Ethnic association studies (DIAMANTE) (cases = 74,124, controls = 824,006) (mean age: cases 55.2 years and controls 52.7 years; proportion of women: cases 49.6% and controls 48.0%) as part of the DIAGRAM consortium, adjusted for study-specific covariates and principal components and corrected for genomic control [33]. Since the UK Biobank is included in the DIAMANTE and the summary statistics for the DIAMANTE without the UK Biobank are not publicly available, we checked, rather than replicated, using the UK Biobank GWAS of T2DM (cases = 20,203, controls = 388,756), adjusted for birth year, sex, and four principal components [29].

The secondary outcomes were CVD risk factors. Genetic associations with adiposity were obtained from a meta-analysis of the UK Biobank and the Genetic Investigation of Anthropometric Traits (GIANT) which has inverse normal transformed body mass index (BMI) (*n* = 778,580), adjusted for age, sex, recruitment center, genotyping batches and ten principal components [34] and inverse normal transformed waist-hip ratio (WHR) (*n* = 694,649) from people of European descent, adjusted for age, age^2^, sex, recruitment center, and genotyping array (54.6% women) [35]. Genetic associations with glycemic traits were obtained from the Meta-Analyses of Glucose and Insulin-related traits Consortium (MAGIC) which has glycosylated hemoglobin (HbA1c) (%) (*n* = 159,940) primarily of European (77%), followed by East Asian (13%), South Asian (5.5%), and African descent (4.5%), adjusted for age, sex, and study-specific covariates [36], as well as fasting glucose (mmol/L) (*n* = 133,010), log-transformed fasting insulin (*n* = 108,557) adjusted for age, study site, and geographic covariates [37] (or if not available, fasting glucose (*n* = 46,186) and fasting insulin (*n* = 38,238) based on the 2010 version) [38] from people mainly of European descent without diabetes (mean age: men 56.9 years, women 55.3 years; 49.7% women). Genetic associations with systolic and diastolic blood pressure (mmHg) were obtained from the UK Biobank GWAS (*n* = 340,159), adjusted for age, age^2^, sex, interactions of sex with age and age^2^, and 20 principal components [32]. Genetic associations with lipids were obtained from the Global Lipids Genetics Consortium (GLGC) which has inverse normal transformed total cholesterol, high-density lipoprotein (HDL)-cholesterol, low-density lipoprotein (LDL)-cholesterol, and triglycerides (*n* = 188,577) from people of European descent, adjusted for age, age^2^, and sex and corrected for genomic control (mean age: 55.2 years; 48.0% women) [39].

### Association of genetically predicted IHD, ischemic stroke, and T2DM with cortisol

#### Genetically predicted IHD, ischemic stroke, and T2DM

SNPs genome-wide significantly (*P* < 5 × 10^−8^) and independently (*r*^2^ < 0.001) associated with IHD, ischemic stroke, and T2DM were used as genetic instrumental variables. These SNPs were based on the summary statistics from CARDIoGRAMplusC4D 1000 Genomes-based GWAS [28], MEGASTROKE (Europeans only) [31], and DIAMANTE [33] respectively. Given CARDIoGRAMplusC4D 1000 Genomes-based GWAS and DIAMANTE include some non-Europeans, we also repeated the analysis using SNPs (*P* < 5 × 10^−8^ and *r*^2^ < 0.001 for IHD and T2DM) from the UK Biobank of white British.

#### Genetic associations with cortisol

Genetic associations with cortisol in people of European descent were obtained from cortisol estimates as in Crawford et al. [19].

#### Statistical analysis

Inverse variance weighting (IVW) with multiplicative random-effects was used to combine SNP-specific Wald estimates (SNP on outcome divided by SNP on exposure), from which odds ratio (OR) or beta coefficients (mean differences) with 95% confidence intervals (CIs) and Cochrane’s *Q*-statistic and *P* value for heterogeneity were presented [40]. As sensitivity analyses to assess horizontal pleiotropy, three complementary methods were used with different assumptions for valid estimates: (i) a weighted median which requires at least 50% of the information to be from valid SNPs; (ii) MR-Egger which allows all SNPs to be invalid on condition that the InSIDE (Instrument Strength Independent of Direct Effect) assumption holds, from which an intercept with *P* < 0.05 indicates the presence of pleiotropy and a higher *I*^2^ value indicates the “no measurement error” assumption holds [41]; and (iii) Mendelian Randomization Pleiotropy RESidual Sum and Outlier (MR-PRESSO) identifies potentially pleiotropic outliers and provides estimates after excluding these outlier SNPs [42].

Specifically, for assessing the associations of cortisol with cardiovascular outcomes, to adjust for multiple comparisons, a Bonferroni-corrected significance level of 0.007 (0.05/7) was considered to account for testing seven traits (i.e., IHD, ischemic stroke, T2DM, adiposity, glycemic traits, blood pressure and lipids). To check for possible confounding, the associations of genetic predictors of cortisol with education, Townsend deprivation index, smoking, alcohol drinking, and physical activity were examined using the UK Biobank. To check for robustness, we repeated the analysis only using cortisol SNPs reaching genome-wide significance (*P* < 5 × 10^−8^). Power calculations were performed for the three primary outcomes (IHD, ischemic stroke and T2DM) [43].

Statistical analyses were conducted using R version 3.5.1 (R Foundation for Statistical Computing, Vienna, Austria) with the MendelianRandomization and MRPRESSO R packages unless specified.

## Results

### Association of genetically predicted cortisol with IHD, ischemic stroke, T2DM, and other CVD risk factors

**Fig. 1:**
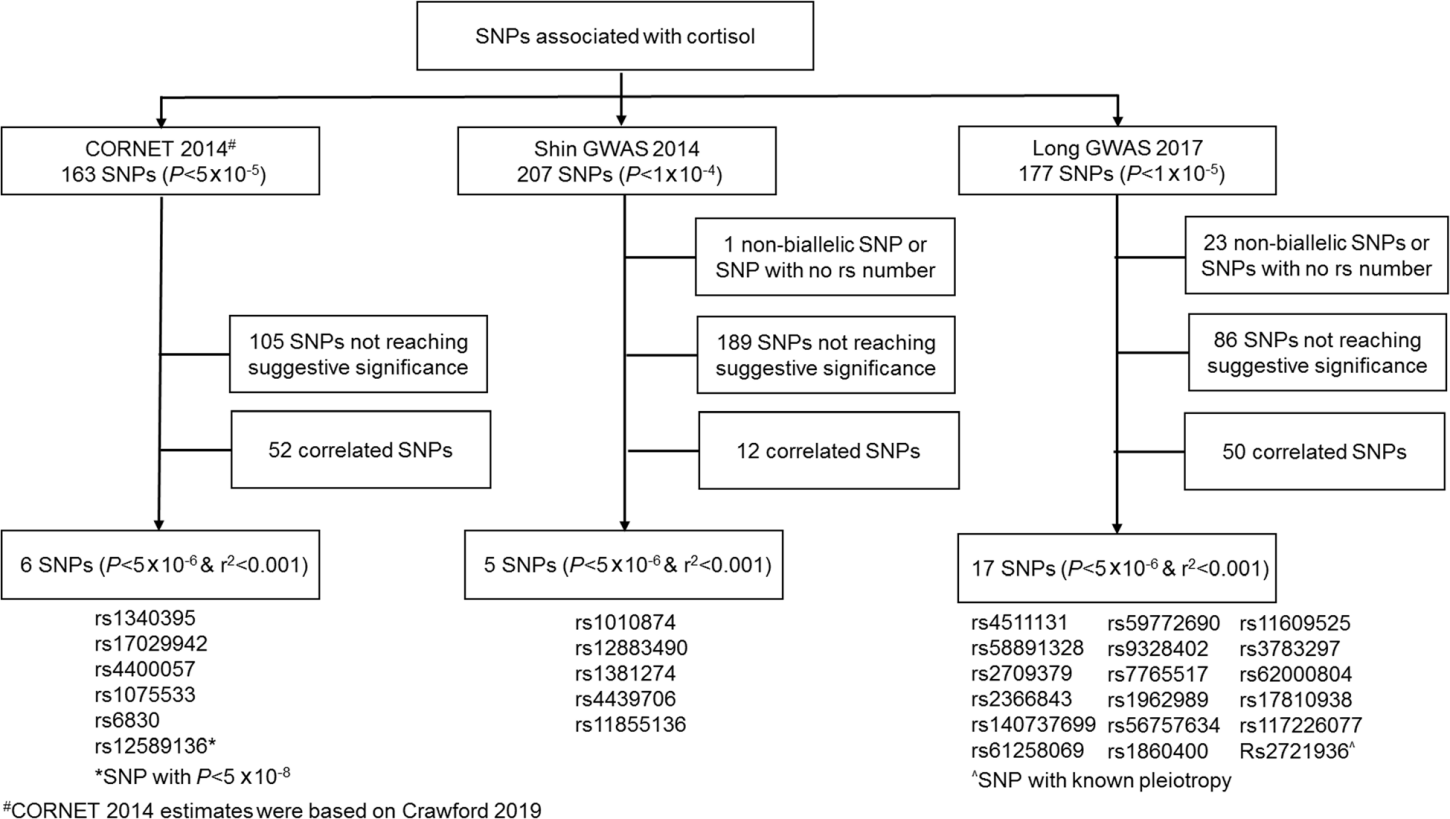
Selection of single nucleotide polymorphisms (SNPs) for Mendelian randomization (MR) analysis of the association of cortisol with ischemic heart disease (IHD), ischemic stroke, type 2 diabetes (T2DM), and cardiovascular disease (CVD) risk factors

A total of 547 SNPs for cortisol were reported by the CORNET, Shin GWAS, and Long GWAS (Fig. 1). Among 163 SNPs from CORNET, 105 SNPs not reaching suggestive significance (*P* ≥ 5 × 10^−6^) and 52 correlated (*r*^2^ ≥ 0.001) SNPs were excluded. Among 207 SNPs from the Shin GWAS, 1 non-biallelic SNP, 189 SNPs not reaching suggestive significance and 12 correlated SNPs were excluded. Among 177 SNPs from the Long GWAS, 22 non-biallelic SNP, 86 SNPs not reaching suggestive significance, and 50 correlated SNPs were excluded. Of the 29 included SNPs, 6 were from the CORNET (overall *F*-statistic = 28.3), 5 from the Shin GWAS (*F* = 23.9), and 18 from the Long GWAS (*F* = 14.9) (Additional file 1: Table S1). Based on known horizontal pleiotropy from PhenoScanner, 1 SNP from Long GWAS (*rs2721936*) associated with phenotypes (body size and composition, erythrocytes, leukocytes, and hematocrit) was excluded in the sensitivity analyses. Genetically predicted cortisol was unrelated to education, Townsend deprivation index, smoking, alcohol drinking, and physical activity (Additional file 1: Table S2).

Table 1 shows that genetically predicted cortisol was not associated with IHD using IVW based on 6 SNPs for cortisol from CORNET and genetic associations with IHD from the CARDIoGRAMplusC4D 1000 Genomes-based GWAS. Sensitivity analyses using a weighted median, MR-Egger, and MR-PRESSO showed similar null findings, with no indication of possible horizontal pleiotropy from the MR-Egger intercept. Replication using the UK Biobank GWAS for IHD showed null results. Similarly, based on 5 SNPs for cortisol from the Shin GWAS and 18 SNPs cortisol from the Long GWAS, genetically predicted cortisol was not associated with IHD.

**Table 1:** Association of genetically predicted cortisol (*P* value< 5 × 10^−6^ and *r*^2^ < 0.001) based on 3 separate data sources (CORtisol NETwork (CORNET) consortium, Shin GWAS, and Long GWAS) with ischemic heart disease (IHD) based on the CARDIoGRAMplusC4D 1000 Genomes-based GWAS (1000 Genomes) with replication based on the UK Biobank using Mendelian randomization (MR) with different methods

Exposure sources	Outcome sources	SNPs	*F-*statistic	Method	Odds ratio	95% CI		*P* value	IVW		MR-Egger	
									Cochran’s *Q*-statistic	*P* value	Intercept *P* value	*I*^2^
CORNET 2014	1000 Genomes	6	28.3	IVW	0.98	0.93	1.03	0.42	2.18	0.82		
				WM	1.00	0.93	1.07	0.95				
				MR-Egger	0.98	0.90	1.06	0.63			0.96	81.5%
				MR-PRESSO	0.98	0.93	1.02	0.28				
	UK Biobank	6	28.3	IVW	0.99	0.93	1.05	0.71	2.29	0.81		
				WM	0.99	0.92	1.07	0.82				
				MR-Egger	0.99	0.89	1.10	0.85			0.96	71.3%
				MR-PRESSO	0.99	0.94	1.04	0.61				
Shin GWAS 2014	1000 Genomes	5	23.9	IVW	0.74	0.47	1.17	0.19	4.52	0.34		
				WM	0.78	0.45	1.35	0.37				
				MR-Egger	0.84	0.32	2.20	0.72			0.77	35.3%
				MR-PRESSO	0.74	0.39	1.41	0.26				
	UK Biobank	5	23.9	IVW	0.96	0.56	1.67	0.89	6.21	0.18		
				WM	1.09	0.61	1.95	0.76				
				MR-Egger	1.58	0.58	4.28	0.37			0.25	9.3%
				MR-PRESSO	0.96	0.44	2.10	0.90				
Long GWAS 2017	1000 Genomes	18	14.9	IVW	1.02	0.99	1.05	0.18	21.43	0.21		
				WM	1.01	0.98	1.05	0.44				
				MR-Egger	1.02	0.96	1.09	0.47			0.91	0%
				MR-PRESSO	1.02	0.99	1.05	0.20				
	UK Biobank	18	14.9	IVW	0.99	0.96	1.01	0.31	15.35	0.57		
				WM	0.99	0.95	1.03	0.57				
				MR-Egger	1.00	0.94	1.05	0.89			0.70	0%
				MR-PRESSO	0.99	0.96	1.01	0.30				

*Abbreviations*: *CI* confidence interval, *IVW* inverse variance weighting, *MR* Mendelian randomization, *SNP* single nucleotide polymorphism, *WM* weighted median

Table 2 shows that genetically predicted cortisol was not associated with ischemic stroke using IVW based on 6 SNPs for cortisol from CORNET and genetic associations with ischemic stroke from MEGASTROKE. Sensitivity analyses using a weighted median, MR-Egger, and MR-PRESSO showed similar null findings, with no indication of possible horizontal pleiotropy from the MR-Egger intercept. Based on 5 SNPs for cortisol from the Shin GWAS, genetically predicted cortisol was associated with lower risk of ischemic stroke using IVW. Sensitivity analyses using a weighted median and MR-PRESSO showed similar associations at a nominal *P* value (*P* < 0.05) but not at Bonferroni-corrected significance (*P* < 0.007) and MR-Egger found no association. The inverse association of cortisol with ischemic stroke was not found based on 18 SNPs cortisol from the Long GWAS.

**Table 2:** Association of genetically predicted cortisol (*P* value< 5 × 10^−6^ and *r*^2^ < 0.001) based on 3 separate data sources (CORtisol NETwork (CORNET) consortium, Shin GWAS, and Long GWAS) with ischemic stroke based on the MEGASTROKE using Mendelian randomization (MR) with different methods

Exposure sources	Outcome sources	SNPs	*F*-statistic	Method	Odds ratio	95% CI		*P* value	IVW		MR-Egger	
									Cochran’s *Q*-statistic	*P* value	Intercept *P* value	*I*^2^
CORNET 2014	MEGASTROKE	6	28.3	IVW	0.99	0.91	1.08	0.83	8.00	0.16		
				WM	0.99	0.91	1.08	0.83				
				MR-Egger	0.95	0.82	1.10	0.46			0.44	73.5%
				MR-PRESSO	0.99	0.88	1.11	0.84				
Shin GWAS 2014	MEGASTROKE	5	23.9	IVW	0.39	0.24	0.64	0.0002	3.15	0.53		
				WM	0.48	0.25	0.93	0.03				
				MR-Egger	0.62	0.25	1.55	0.31			0.25	14.5%
				MR-PRESSO	0.39	0.21	0.73	0.01				
Long GWAS 2017	MEGASTROKE	18	14.9	IVW	1.00	0.97	1.03	0.90	8.93	0.94		
				WM	1.00	0.96	1.04	0.88				
				MR-Egger	1.02	0.96	1.09	0.47			0.45	0%
				MR-PRESSO	1.00	0.98	1.02	0.86				

*Abbreviations*: *CI* confidence interval, *IVW* inverse variance weighting, *MR* Mendelian randomization, *SNP* single nucleotide polymorphism, *WM* weighted median

Table 3 shows that genetically predicted cortisol was not associated with T2DM using IVW based on 6 SNPs for cortisol from CORNET and genetic associations with T2DM from DIAMANTE. Sensitivity analyses using a weighted median, MR-Egger, and MR-PRESSO showed similar null findings, with no indication of possible horizontal pleiotropy from the MR-Egger intercept. Checking using the UK Biobank GWAS for T2DM showed null results. Similarly, based on 5 SNPs for cortisol from the Shin GWAS and 18 SNPs cortisol from the Long GWAS, genetically predicted cortisol was not associated with T2DM.

**Table 3:** Association of genetically predicted cortisol (*P* value< 5 × 10^−6^ and *r*^2^ < 0.001) based on 3 separate data sources (CORtisol NETwork (CORNET) consortium, Shin GWAS, and Long GWAS) with type 2 diabetes (T2DM) based on the DIAbetes Meta-ANalysis of Trans-Ethnic association studies (DIAMANTE) with checking based on the UK Biobank using Mendelian randomization (MR) with different methods

Exposure sources	Outcome sources	SNPs	*F*-statistic	Method	Odds ratio	95% CI		*P* value	IVW		MR-Egger	
									Cochran’s *Q*-statistic	*P* value	Intercept *P* value	*I*^2^
CORNET 2014	DIAMANTE	6	28.3	IVW	1.00	0.96	1.04	0.92	4.23	0.52		
				WM	0.99	0.94	1.05	0.80				
				MR-Egger	0.99	0.92	1.06	0.72			0.70	72.9%
				MR-PRESSO	1.00	0.95	1.05	0.92				
	UK Biobank	6	28.3	IVW	1.00	0.91	1.10	0.999	7.48	0.19		
				WM	1.02	0.92	1.13	0.69				
				MR-Egger	1.11	0.98	1.26	0.10			0.04	71.4%
				MR-PRESSO	1.00	0.88	1.13	0.999				
Shin GWAS 2014	DIAMANTE	5	23.9	IVW	0.68	0.46	1.02	0.07	6.93	0.14		
				WM	0.70	0.46	1.06	0.09				
				MR-Egger	0.70	0.29	1.69	0.43			0.96	3.2%
				MR-PRESSO	0.68	0.39	1.21	0.14				
	UK Biobank	5	23.9	IVW	0.68	0.34	1.39	0.29	6.70	0.15		
				WM	0.71	0.34	1.48	0.36				
				MR-Egger	1.13	0.28	4.52	0.86			0.40	10.6%
				MR-PRESSO	0.68	0.25	1.86	0.35				
Long GWAS 2017	DIAMANTE	18	14.9	IVW	1.00	0.97	1.03	0.95	57.78	< 0.0001		
				WM	0.99	0.96	1.02	0.33				
				MR-Egger	0.99	0.92	1.05	0.69			0.67	0%
				MR-PRESSO^a^	0.99	0.96	1.01	0.33				
	UK Biobank	18	14.9	IVW	1.01	0.97	1.05	0.60	24.35	0.11		
				WM	1.01	0.96	1.05	0.74				
				MR-Egger	0.97	0.90	1.05	0.43			0.24	0%
				MR-PRESSO	1.01	0.97	1.05	0.61				

*Abbreviations*: *CI* confidence interval, *IVW* inverse variance weighting, *MR* Mendelian randomization, *SNP* single nucleotide polymorphism, *WM* weighted median

^a^MR-PRESSO estimate was obtained by excluding 1 outlier (*rs117226077*)

Repeating the analysis after excluding 1 SNP from the Long GWAS with potential horizontal pleiotropy also showed genetically predicted cortisol was not associated with IHD, ischemic stroke, or T2DM (Additional file 1: Table S3). Using 1 SNP reaching genome-wide significance (*rs12589136* from the CORNET) also found a similarly null association of cortisol with IHD, ischemic stroke or T2DM (Additional file 1: Table S4). In the secondary analyses, firstly, based on *P* value based effect size corrected for sample overlap, using 29 SNPs from the three GWAS, genetically predicted cortisol was not associated with IHD, ischemic stroke or T2DM (Additional file 2: Tables S1-S4). Secondly, based on 23 SNPs with all estimates from Crawford et al. (of which 6 SNPs unavailable in Crawford), null associations were also found (Additional file 3: Tables S1-S4). Thirdly, based on 42 SNPs identified from the meta-analysis of all SNPs available in the three cortisol GWAS, the null associations of cortisol with IHD, ischemic stroke, or T2DM remain unchanged (Additional file 4: Tables S1-S4).

Table 4 shows that genetically predicted cortisol was not associated with BMI, WHR, HbA1c, fasting glucose, fasting insulin, systolic or diastolic blood pressure, total cholesterol, HDL-cholesterol, LDL-cholesterol, or triglycerides using IVW based on 6 SNPs for cortisol from CORNET. Sensitivity analyses using a weighted median, MR-Egger, and MR-PRESSO showed similar patterns of association. An inverse association of cortisol with systolic blood pressure was found using MR-Egger, but possible horizontal pleiotropy cannot be ruled out (intercept *P* value = 0.001). Also, a weighted median showed null association and MR-PRESSO showed a null association after excluding one SNP outlier (*rs6830*).

**Table 4:** Association of genetically predicted cortisol (*P* value< 5 × 10^−6^ and *r*^2^ < 0.001) based on the CORtisol NETwork (CORNET) consortium, with cardiovascular disease (CVD) risk factors (adiposity based on the Genetic Investigation of Anthropometric Traits (GIANT), glycemic traits based on the Meta-Analyses of Glucose and Insulin-related traits Consortium (MAGIC), blood pressure based on the UK Biobank, and lipids based on the Global Lipids Genetics Consortium (GLGC)) using Mendelian randomization (MR) with different methods

Outcomes	Sources	SNPs	*F*-statistic	Method	Mean difference	95% CI		*P* value	IVW		MR-Egger	
									Cochran’s *Q*-statistic	*P* value	Intercept *P* value	*I*^2^
Adiposity												
BMI	GIANT	6	28.3	IVW	− 0.001	− 0.01	0.01	0.85	2.37	0.80		
				WM	− 0.003	− 0.02	0.01	0.72				
				MR-Egger	0.01	− 0.01	0.03	0.55			0.36	71.3%
				MR-PRESSO	− 0.001	− 0.01	0.01	0.80				
WHR	GIANT	6	28.3	IVW	− 0.01	− 0.02	0.01	0.26	7.55	0.18		
				WM	− 0.01	− 0.02	0.01	0.53				
				MR-Egger	0.003	− 0.02	0.03	0.76			0.17	71.8%
				MR-PRESSO	− 0.01	− 0.03	0.01	0.31				
Glycemic traits												
HbA1c	MAGIC	6	28.3	IVW	− 0.01	− 0.03	0.01	0.46	5.89	0.32		
				WM	− 0.005	− 0.03	0.02	0.72				
				MR-Egger	0.004	− 0.03	0.04	0.80			0.39	27.6%
				MR-PRESSO	− 0.01	− 0.03	0.02	0.49				
Glucose	MAGIC	6	28.3	IVW	0.02	− 0.004	0.05	0.10	4.64	0.46		
				WM	0.02	− 0.02	0.05	0.31				
				MR-Egger	0.04	− 0.004	0.08	0.08			0.33	73.1%
				MR-PRESSO	0.02	− 0.01	0.05	0.15				
Insulin	MAGIC	6	28.3	IVW	0.01	− 0.02	0.04	0.45	3.12	0.68		
				WM	0.01	− 0.02	0.05	0.51				
				MR-Egger	0.01	− 0.04	0.05	0.77			0.82	73.1%
				MR-PRESSO	0.01	− 0.02	0.04	0.38				
Blood pressure												
Systolic BP	UK Biobank	6	28.3	IVW	0.01	− 0.03	0.04	0.71	21.11	0.001		
				WM	− 0.002	− 0.02	0.02	0.86				
				MR-Egger	− 0.03	− 0.06	− 0.003	0.03			0.001	71.5%
				MR-PRESSO^a^	− 0.001	− 0.02	0.02	0.93				
Diastolic BP	UK Biobank	6	28.3	IVW	0.001	− 0.03	0.03	0.93	12.87	0.02		
				WM	0.01	− 0.01	0.03	0.52				
				MR-Egger	− 0.02	− 0.06	0.01	0.18			0.07	71.5%
				MR-PRESSO	0.001	− 0.03	0.04	0.93				
Lipids												
Total Cholesterol	GLGC	6	28.3	IVW	− 0.02	− 0.06	0.01	0.21	5.39	0.37		
				WM	− 0.04	− 0.08	0.01	0.12				
				MR-Egger	− 0.04	− 0.11	0.02	0.20			0.48	74.7%
				MR-PRESSO	− 0.02	− 0.07	0.03	0.26				
HDL-Cholesterol	GLGC	6	28.3	IVW	0.01	− 0.04	0.05	0.80	9.36	0.10		
				WM	− 0.003	− 0.05	0.04	0.89				
				MR-Egger	− 0.04	− 0.10	0.03	0.28			0.11	75.1%
				MR-PRESSO	0.01	− 0.06	0.07	0.81				
LDL-Cholesterol	GLGC	6	28.3	IVW	− 0.03	− 0.07	0.003	0.07	4.20	0.52		
				WM	− 0.04	− 0.09	0.005	0.08				
				MR-Egger	− 0.04	− 0.10	0.02	0.19			0.78	75.4%
				MR-PRESSO	− 0.03	− 0.08	0.01	0.11				
Triglycerides	GLGC	6	28.3	IVW	− 0.01	− 0.04	0.03	0.70	2.16	0.83		
				WM	− 0.01	− 0.05	0.03	0.69				
				MR-Egger	− 0.01	− 0.06	0.04	0.72			0.88	75.8%
				MR-PRESSO	− 0.01	− 0.04	0.02	0.59				

*Abbreviations*: *BP* blood pressure, *CI* confidence interval, *HDL* high-density lipoprotein, *LDL* low-density lipoprotein, *IVW* inverse variance weighting, *MR* Mendelian randomization, *SNP* single nucleotide polymorphism, *WM* weighted median

^a^MR-PRESSO estimate was obtained by excluding 1 outlier (*rs6830*)

Power calculations showed that this study based on 6 SNPs for cortisol from the CORNET (*R*^2^ = 0.014) had 80% power at 0.05 significance level to detect OR = 1.12 for IHD, OR = 1.13 for ischemic stroke, and OR = 1.09 for T2DM.

### Association of genetically predicted IHD, ischemic stroke, and T2DM cortisol with cortisol

A total of 41 SNPs for IHD from CARDIoGRAMplusC4D 1000 Genomes-based GWAS, 10 SNPs for ischemic stroke from MEGASTROKE, and 202 SNPs for T2DM from DIAMANTE reached genome-wide significance and were independent. Among 41 SNPs for IHD, 22 original SNPs were available in the cortisol GWAS from Crawford et al. [19]; 17 proxy SNPs were identified based on LDLink and 2 had no proxy SNPs. Among 10 SNPs for ischemic stroke, 7 original SNPs were available in cortisol GWAS; 2 proxy SNPs were identified and 1 had no proxy SNP. Among 202 SNPs for T2DM, 95 original SNPs were available in cortisol GWAS; 71 proxy SNPs were identified, 34 had no proxy SNPs, and 2 were non-biallelic SNPs. As such, 39 SNPs were included for IHD (overall *F*-statistic = 61.3), 9 SNPs for ischemic stroke (*F* = 40.8), and 166 for T2DM (*F* = 40.8).

Table 5 shows that genetically predicted IHD was not associated with cortisol using IVW based on 39 SNPs for IHD from CARDIoGRAMplusC4D 1000 Genomes-based GWAS and genetic associations with cortisol from Crawford et al. [19]. Sensitivity analyses using a weighted median, MR-Egger, and MR-PRESSO showed similar null findings, with no indication of possible horizontal pleiotropy from the MR-Egger intercept. Similarly, based on 9 SNPs for ischemic stroke from MEGASTROKE and 166 SNPs for T2DM from DIAMANTE, neither genetically predicted ischemic stroke nor genetically predicted T2DM was associated with cortisol. Using SNPs for IHD and T2DM from the UK Biobank white British also showed null results on cortisol (Table 6).

**Table 5:** Association of genetically predicted ischemic heart disease (IHD) (*P* value< 5 × 10^−8^ and *r*^2^ < 0.001) based on the CARDIoGRAMplusC4D 1000 Genomes-based GWAS (1000 Genomes), genetically predicted ischemic stroke based on the MEGASTROKE, and genetically predicted type 2 diabetes (T2DM) based on the DIAbetes Meta-ANalysis of Trans-Ethnic association studies (DIAMANTE) with cortisol based on the Crawford et al. study [19] using Mendelian randomization (MR) with different methods

Exposure sources	Outcome sources	SNPs	*F*-statistic	Method	Beta	95% CI		*P* value	IVW		MR-Egger	
									Cochran’s *Q*-statistic	*P* value	Intercept *P* value	*I*^2^
IHD	Cortisol	39	61.3	IVW	− 0.03	− 0.08	0.03	0.38	37.37	0.50		
(1000 Genomes)	(Crawford 2019)			WM	− 0.08	− 0.17	0.01	0.08				
				MR-Egger	− 0.01	− 0.16	0.13	0.88			0.83	96.1%
				MR-PRESSO	− 0.03	− 0.09	0.03	0.38				
Ischemic stroke	Cortisol	9	40.8	IVW	− 0.06	− 0.19	0.07	0.35	7.10	0.53		
(MEGASTROKE)	(Crawford 2019)			WM	− 0.004	− 0.17	0.17	0.97				
				MR-Egger	− 0.45	− 1.42	0.53	0.37			0.44	0%
				MR-PRESSO	− 0.06	− 0.20	0.08	0.35				
T2DM	Cortisol	166	80.8	IVW	0.01	− 0.03	0.04	0.60	163.50	0.52		
(DIAMANTE)	(Crawford 2019)			WM	0.001	− 0.07	0.07	0.99				
				MR-Egger	− 0.002	− 0.08	0.07	0.96			0.75	96.7%
				MR-PRESSO	0.01	− 0.03	0.04	0.60				

*Abbreviations*: *CI* confidence interval, *IVW* inverse variance weighting, *MR* Mendelian randomization, *SNP* single nucleotide polymorphism, *WM* weighted median

**Table 6:** Association of genetically predicted ischemic heart disease (IHD) and genetically predicted type 2 diabetes (T2DM) (*P* value< 5 × 10^−8^ and *r*^2^ < 0.001) based on the UK Biobank with cortisol based on the Crawford et al. study [19] using Mendelian randomization (MR) with different methods

Exposure sources	Outcome sources	SNPs	*F*-statistic	Method	Beta	95% CI		*P* value	IVW		MR-Egger	
									Cochran’s *Q*-statistic	*P* value	Intercept *P* value	*I*^2^
IHD	Cortisol	32	60.2	IVW	− 0.001	− 0.07	0.07	0.97	30.18	0.51		
(UK Biobank)	(Crawford 2019)			WM	0.001	− 0.10	0.11	0.99				
				MR-Egger	− 0.03	− 0.22	0.16	0.78			0.78	94.9%
				MR-PRESSO	− 0.001	− 0.07	0.07	0.97				
T2DM	Cortisol	35	73.4	IVW	− 0.004	− 0.06	0.05	0.88	46.44	0.08		
(UK Biobank)	(Crawford 2019)			WM	0.002	− 0.07	0.08	0.97				
				MR-Egger	0.02	− 0.12	0.15	0.81			0.74	96.6%
				MR-PRESSO	− 0.004	− 0.06	0.05	0.88				

*Abbreviations*: *CI* confidence interval, *IVW* inverse variance weighting, *MR* Mendelian randomization, *SNP* single nucleotide polymorphism, *WM* weighted median

## Discussion

This two-sample MR study does not suggest cortisol plays a major role in cardiovascular disease given genetically predicted cortisol was unrelated to IHD, ischemic stroke, T2DM or CVD risk factors (adiposity, glycemic traits, blood pressure, and lipids) as well as genetically predicted IHD, ischemic stroke, and T2DM being unrelated to cortisol. Cortisol was associated with a lower risk of ischemic stroke in some sensitivity analyses, but not the main analyses. Replication using stronger or functionally relevant SNPs for cortisol is warranted. Nonetheless, these MR findings raise a question about the relevance of cortisol-related pathways to cardiovascular health.

Some limitations have to be considered. First, we used independent SNPs that predict cortisol based on suggestive significance (*P* < 5 × 10^−6^) given only one SNP reached genome-wide significance and functionally relevant SNPs are not well-established for cortisol. Weak instrument bias is possible, particularly as the genetic instruments selected from each of the three cortisol GWAS did not overlap. However, analysis using the genetic instruments from all three cortisol GWAS with the estimates taken from the largest study, i.e., Crawford, gave similarly null results. Also, our *F*-statistic > 10 lowers the possibility of weak instrument bias [44] and this study is powered to detect small effect sizes (OR ranging from 1.09 to 1.13) which would be able to detect the effect size generally reported in prospective studies (OR ranging from 1.22 to 2.56) [9, 14, 15]. Moreover, similar null results were found when using the only SNP reaching genome-wide significance. Secondary analyses using SNPs originally identified from each of the three cortisol GWAS or identified based on the meta-analysis of the three cortisol GWAS also gave null results. Conversely, we used independent SNPs that predict IHD, ischemic stroke, and T2DM based on genome-wide significance. Considering the cortisol GWAS is less extensively genotyped, proxy SNPs were identified whenever available based on LDLink but several SNPs without proxies could not be incorporated. However, our *F*-statistic for all these SNPs > 10 lowers the possibility of weak instrument bias. Second, excluding known pleiotropic SNPs gave similarly null findings for IHD, ischemic stroke, and T2DM. Some pleiotropic effects may arguably be potential mediators (e.g., BMI might be considered as both vertical and horizontal pleiotropy) such that removing these SNPs might not produce robust causal estimates. Nonetheless, the null findings remain unchanged before and after excluding any potentially pleiotropic SNPs and after considering any statistical evidence of unknown pleiotropy based on the weighted median, MR-Egger, and MR-PRESSO [42]. Third, a polygenic risk score would have generally high predictive power using a larger number of SNPs based on a less stringent threshold of significance (e.g., *P* < 5 × 10^−5^) [45], but requires individual level data, which are not publicly available. Although it helps identify putative relationships, high false-positive rates due to horizontal pleiotropy are possible and hence further investigation using MR methods has been proposed [46]. As such, this two-sample MR study allows better assessment of any horizontal pleiotropy. Replication using a larger GWAS of cortisol in the future is needed given existing GWAS are relatively small and relevant SNPs may not have been fully identified. Fourth, selection bias might have created false nulls for late onset conditions that share etiology with common conditions that caused death prior to recruitment [47]. Considering IHD is a relatively early-onset disease, MR estimates for IHD may be less subject to survival bias than later-onset diseases, but they were also null. Fifth, MR estimates reflect lifetime differences in exposure (cortisol) [16]; however, the effects of cortisol are not known to change with age [48]. This MR study was based on three GWAS of morning cortisol. Higher evening cortisol could be more relevant to CVD risk as proposed by Cohen and McEwen [49, 50]. However, no GWAS of evening cortisol is available and there is no agreement as to which measure of cortisol is most causal of CVD risk. Furthermore, this MR study cannot distinguish acute from chronic exposure to stress or cortisol and their short-term versus long-term effects. It also cannot assess whether cumulative or critical period exposures matter and if there is any critical timing of exposure. Sixth, the genetic effects of cortisol might be buffered by a compensatory mechanism, although whether or how such developmental canalization operates is unknown [51]. Finally, the applicability of our findings based on Caucasians to other populations including Chinese needs further investigation, considering the relevance of a causal factor may vary by setting.

Our MR findings are inconsistent with previous observational studies [9–15, 52]. However, these studies are primarily from settings where stress or cortisol is associated with socioeconomic position (SEP), hence are subject to residual or unmeasured confounding by socioeconomic related attributes, given lower SEP is usually associated with higher cortisol, which may explain the link with poor health [53]. In addition, cortisol may be a symptom of underlying illness or prevalent diseases [54]. Further, stress has other effects that could be protective because stress affects other hormones such as testosterone [6], which is emerging as relevant to IHD [55, 56]. Our MR findings on cortisol are more consistent with a previous MR study showing null effects of subjective well-being on IHD and CVD risk factors although higher BMI may affect subjective well-being [17]. Similarly, using a Bayesian network to prune indirect links suggested that depression-related co-morbidities, such as T2DM or other CVD risk factors, may be unrelated to depression [18]. Alternatively, genetically predicted cortisol examined in MR studies may differ from stress-induced cortisol considered in observational studies. In response to stress, elevated cortisol may co-occur with other biological and behavioral changes such that stress-induced cortisol may be part of a different pathway linking stress to CVD.

To our knowledge, few previous MR studies have investigated the role of cortisol. A recent MR study suggested genetically predicted cortisol was positively associated with IHD (OR 1.06, 95% CI 0.98 to 1.15) using IVW based on 3 SNPs (*rs12589136*, *rs2749527*, *rs11621961*) with some LD (*r*^2^ < 0.3) from CORNET [19] and genetic associations with IHD from a meta-analysis of UK Biobank and CARDIoGRAMplusC4D [57]. The effect size was smaller than its previous poster abstract (OR 1.27, 95% CI 1.01 to 1.60) based on the same SNPs [58] and CARDIoGRAM [59]. However, such effect on IHD did not replicate using weighted generalized linear regression or IVW with a correlation matrix for correlated SNPs [60] when applying these SNPs to the CARDIoGRAM [59], CARDIoGRAMplusC4D 1000 Genomes-based GWAS [28], or meta-analysis of UK Biobank and CARDIoGRAMplusC4D [57] (Additional file 1: Table S5). A recent MR study showed genetically predicted cortisol positively associated with IHD [20], based on 2 independent SNPs using one-sample MR among healthy participants and patients with suspected or confirmed IHD, which may be subject to selection bias given prior deaths and/or healthy controls were excluded from the study [61]. No effect on IHD was found when applying these SNPs to the CARDIoGRAMplusC4D 1000 Genomes-based GWAS [28] or UK Biobank GWAS of IHD [29] (Additional file 1: Table S6).

This MR study should be confirmed or refuted using stronger genetic instruments when a larger GWAS on cortisol becomes available. Nevertheless, our findings may stimulate discussion as to the relevance and importance of the HPA axis pathway, particularly via cortisol, to CVD and more broadly concerning the link between mental and physical health [62].

## Conclusion

Our study found no evidence that cortisol causes IHD, ischemic stroke, T2DM, or CVD risk factors, but cannot exclude a small effect. This study also found no evidence that IHD, ischemic stroke, and T2DM affect cortisol. The role of cortisol-related pathway to CVD is cast into doubt. Better understanding of alternative biological and behavioral pathways underlying the relation between stress and CVD, including inflammation, stress-coping behaviors, and mental health would provide insights on developing more effective CVD interventions, especially stress in daily life may hinder adherence to existing interventions (such as lifestyle modification and cholesterol-lowering medication).

## Supplementary Information

**Additional file 1:** **Table S1.** Single nucleotide polymorphisms (SNPs) considerably (*P*-value< 5 × 10^−6^) and independently (r^2^ < 0.001) associated with cortisol (total SNPs = 29). **Table S2.** Association of genetically predicted cortisol (*P*-value< 5 × 10^−6^ and r^2^ < 0.001) based on SNPs from the CORtisol NETwork (CORNET) consortium with socio-economic position (education, and Townsend deprivation index) and lifestyle (smoking, alcohol drinking, and physical activity) from the UK Biobank using Mendelian randomization (MR) with different methods. **Table S3.** Association of genetically predicted cortisol based on one single nucleotide polymorphism (SNP) reaching genome-wide significance (*P*-value< 5 × 10^−6^ and r^2^ < 0.001) from Long GWAS after excluding one SNP with known horizontal pleiotropy (*rs2721936*) with ischemic heart disease (IHD) based on the CARDIoGRAMplusC4D 1000 Genomes-based GWAS (1000 Genomes) with replication based on the UK Biobank, ischemic stroke based on the MEGASTROKE and type 2 diabetes (T2DM) based on the DIAbetes Meta-ANalysis of Trans-Ethnic association studies (DIAMANTE) with checking based on the UK Biobank using Mendelian randomization (MR)^a^. **Table S4.** Association of genetically predicted cortisol based on one single nucleotide polymorphism (SNP) reaching genome-wide significance (*rs12589136*) (*P*-value< 5 × 10^−8^ and r^2^ < 0.001) from CORtisol NETwork (CORNET) consortium with ischemic heart disease (IHD) based on the CARDIoGRAMplusC4D 1000 Genomes-based GWAS (1000 Genomes) with replication based on the UK Biobank, ischemic stroke based on the MEGASTROKE and type 2 diabetes (T2DM) based on the DIAbetes Meta-ANalysis of Trans-Ethnic association studies (DIAMANTE) with checking based on the UK Biobank using Mendelian randomization (MR)^a^. **Table S5**. Association of genetically predicted cortisol based on 3 correlated SNPs used in Crawford et al. studies^a^ with ischemic heart disease based on the CARDIoGRAM (original dataset used in Crawford et al. [58] poster abstract), CARDIoGRAMplusC4D 1000 Genomes-based GWAS (1000 Genomes), and a meta-analysis of UK Biobank and CARDIoGRAMplusC4D (CAD_META) (original dataset used in Crawford et al. [19] paper) using Mendelian randomization (MR) with different methods. **Table S6**. Association of genetically predicted cortisol based on 2 independent SNPs used in Pott et al. study^a^ with ischemic heart disease based on the CARDIoGRAMplusC4D 1000 Genomes-based GWAS (1000 Genomes) with replication based on the UK Biobank using Mendelian randomization (MR) with different methods.

**Additional file 2: Table S1.** Single nucleotide polymorphisms (SNPs) considerably (*P*-value< 5 × 10^−6^) and independently (r^2^ < 0.001) associated with cortisol from three data sources (CORtisol NETwork (CORNET) consortium, Shin GWAS and Long GWAS) using *P*-value based effect size with sample overlap correction^a^ (total SNPs = 29). **Table S2.** Association of genetically predicted cortisol (*P*-value< 5 × 10^−6^ and r^2^ < 0.001) based on single nucleotide polymorphisms (SNPs) from three data sources (CORtisol NETwork (CORNET) consortium, Shin GWAS and Long GWAS) using *p*-value based effect size with sample overlap correction with ischemic heart disease (IHD) based on the CARDIoGRAMplusC4D 1000 Genomes-based GWAS (1000 Genomes) with replication based on the UK Biobank using Mendelian randomization (MR) with different methods. **Table S3.** Association of genetically predicted cortisol (*P*-value< 5 × 10^−6^ and r^2^ < 0.001) based on single nucleotide polymorphisms (SNPs) from three data sources (CORtisol NETwork (CORNET) consortium, Shin GWAS and Long GWAS) using p-value based effect size with sample overlap correction with ischemic stroke based on the MEGASTROKE using Mendelian randomization (MR) with different methods. **Table S4**. Association of genetically predicted cortisol (*P*-value< 5 × 10^−6^ and r^2^ < 0.001) based on single nucleotide polymorphisms (SNPs) from three data sources (CORtisol NETwork (CORNET) consortium, Shin GWAS and Long GWAS) using p-value based effect size with sample overlap correction with type 2 diabetes (T2DM) based on the DIAbetes Meta-ANalysis of Trans-Ethnic association studies (DIAMANTE) with checking based on the UK Biobank using Mendelian randomization (MR) with different methods.

**Additional file 3: Table S1**. Single nucleotide polymorphisms (SNPs) considerably (*P*-value< 5 × 10^−6^) and independently (r^2^ < 0.001) associated with cortisol from three data sources (CORtisol NETwork (CORNET) consortium, Shin GWAS and Long GWAS) using estimates based on Crawford et al. Eur J Endocrinol. 2019^a^ (total SNPs = 23). **Table S2**. Association of genetically predicted cortisol (*P*-value< 5 × 10^−6^ and r^2^ < 0.001) based on single nucleotide polymorphisms (SNPs) from three data sources (CORtisol NETwork (CORNET) consortium, Shin GWAS and Long GWAS) using estimates based on Crawford et al. [19] study with ischemic heart disease (IHD) based on the CARDIoGRAMplusC4D 1000 Genomes-based GWAS (1000 Genomes) with replication based on the UK Biobank using Mendelian randomization (MR) with different methods. **Table S3.** Association of genetically predicted cortisol (*P*-value< 5 × 10^−6^ and r^2^ < 0.001) based on single nucleotide polymorphisms (SNPs) from three data sources (CORtisol NETwork (CORNET) consortium, Shin GWAS and Long GWAS) using estimates based on Crawford et al. [19] study with ischemic stroke based on the MEGASTROKE using Mendelian randomization (MR) with different methods. **Table S4.** Association of genetically predicted cortisol (*P*-value< 5 × 10^−6^ and r^2^ < 0.001) based on single nucleotide polymorphisms (SNPs) from three data sources (CORtisol NETwork (CORNET) consortium, Shin GWAS and Long GWAS) using estimates based on Crawford et al. [19] study with type 2 diabetes (T2DM) based on the DIAbetes Meta-ANalysis of Trans-Ethnic association studies (DIAMANTE) with checking based on the UK Biobank using Mendelian randomization (MR) with different methods.

**Additional file 4: Table S1.** Single nucleotide polymorphisms (SNPs) considerably (*P*-value< 5 × 10^−6^) and independently (r^2^ < 0.001) associated with cortisol from a meta-analysis across all SNPs available in three data sources (CORtisol NETwork (CORNET) consortium, Shin GWAS and Long GWAS) using *P*-value based effect size with sample overlap correction^a^ (total SNPs = 42). **Table S2.** Association of genetically predicted cortisol (*P*-value< 5 × 10^−6^ and r^2^ < 0.001) based on single nucleotide polymorphisms (SNPs) from a meta-analysis across all SNPs available in three data sources (CORtisol NETwork (CORNET) consortium, Shin GWAS and Long GWAS) using *p*-value based effect size with sample overlap correction with ischemic heart disease (IHD) based on the CARDIoGRAMplusC4D 1000 Genomes-based GWAS (1000 Genomes) with replication based on the UK Biobank using Mendelian randomization (MR) with different methods. **Table S3.** Association of genetically predicted cortisol (*P*-value< 5 × 10^−6^ and r^2^ < 0.001) based on single nucleotide polymorphisms (SNPs) from a meta-analysis across all SNPs available in three data sources (CORtisol NETwork (CORNET) consortium, Shin GWAS and Long GWAS) using p-value based effect size with sample overlap correction with ischemic stroke based on the MEGASTROKE using Mendelian randomization (MR) with different methods. **Table S4.** Association of genetically predicted cortisol (*P*-value< 5 × 10^−6^ and r^2^ < 0.001) based on single nucleotide polymorphisms (SNPs) from a meta-analysis across all SNPs available in three data sources (CORtisol NETwork (CORNET) consortium, Shin GWAS and Long GWAS) using p-value based effect size with sample overlap correction with type 2 diabetes (T2DM) based on the DIAbetes Meta-ANalysis of Trans-Ethnic association studies (DIAMANTE) with checking based on the UK Biobank using Mendelian randomization (MR) with different methods.

## Data Availability

The datasets used and/or analyzed during the current study are publicly available and accessible.

## Abbreviations

CVD: Cardiovascular disease
GWAS: Genome-wide association study
IVW: Inverse variance weighting
IHD: Ischemic heart disease
LD: Linkage disequilibrium
MR: Mendelian randomization
SNP: Single nucleotide polymorphism
T2DM: Type 2 diabetes mellitus
