# Supplementary material for: The role of cortisol in ischemic heart disease, ischemic stroke, type 2 diabetes, and cardiovascular disease risk factors: a bi-directional Mendelian randomization study
Source: BMC Med. 2020 Nov 27;18:363. doi: 10.1186/s12916-020-01831-3 (PMC7694946; doi:10.1186/s12916-020-01831-3)
Supplement: Supplementary file 1 — Additional file 1: Table S1. Single nucleotide polymorphisms (SNPs) considerably (P-value< 5 × 10^−6^) and independently (r2 < 0.001) associated with cortisol (total SNPs = 29). Table S2. Association of genetically predicted cortisol (P-value< 5 × 10^−6^ and r2 < 0.001) based on SNPs from the CORtisol NETwork (CORNET) consortium with socio-economic position (education, and Townsend deprivation index) and lifestyle (smoking, alcohol drinking, and physical activity) from the UK Biobank using Mendelian randomization (MR) with different methods. Table S3. Association of genetically predicted cortisol based on one single nucleotide polymorphism (SNP) reaching genome-wide significance (P-value< 5 × 10^−6^ and r2 < 0.001) from Long GWAS after excluding one SNP with known horizontal pleiotropy (rs2721936) with ischemic heart disease (IHD) based on the CARDIoGRAMplusC4D 1000 Genomes-based GWAS (1000 Genomes) with replication based on the UK Biobank, ischemic stroke based on the MEGASTROKE and type 2 diabetes (T2DM) based on the DIAbetes Meta-ANalysis of Trans-Ethnic association studies (DIAMANTE) with checking based on the UK Biobank using Mendelian randomization (MR)a. Table S4. Association of genetically predicted cortisol based on one single nucleotide polymorphism (SNP) reaching genome-wide significance (rs12589136) (P-value< 5 × 10^−8^ and r2 < 0.001) from CORtisol NETwork (CORNET) consortium with ischemic heart disease (IHD) based on the CARDIoGRAMplusC4D 1000 Genomes-based GWAS (1000 Genomes) with replication based on the UK Biobank, ischemic stroke based on the MEGASTROKE and type 2 diabetes (T2DM) based on the DIAbetes Meta-ANalysis of Trans-Ethnic association studies (DIAMANTE) with checking based on the UK Biobank using Mendelian randomization (MR)a. Table S5. Association of genetically predicted cortisol based on 3 correlated SNPs used in Crawford et al. studiesa with ischemic heart disease based on the CARDIoGRAM (original dataset used in Crawford et al. [58] poster abs [file 12916_2020_1831_MOESM1_ESM.docx]

**Additional file 1**

Table S1. Single nucleotide polymorphisms (SNPs) considerably (*P*-value<5x10^-6^) and independently (r^2^<0.001) associated with cortisol (total SNPs=29)

| Source^a^ | SNP | Chr | Position | Effect  allele | Other  allele | EAF | Beta | SD | *P*-value |
| --- | --- | --- | --- | --- | --- | --- | --- | --- | --- |
| CORNET 2014 | rs1340395 | 1 | 102662715 | T | C | 0.93 | -0.13 | 0.03 | 1.09x10^-6^ |
|  | rs17029942 | 3 | 3320289 | G | A | 0.97 | -0.65 | 0.13 | 3.10x10^-7^ |
|  | rs4400057 | 4 | 58712526 | A | G | 0.91 | -0.32 | 0.07 | 9.46x10^-7^ |
|  | rs1075533 | 11 | 102963776 | G | A | 0.96 | 0.17 | 0.03 | 7.74x10^-7^ |
|  | rs6830 | 14 | 73238184 | G | A | 0.68 | 0.06 | 0.01 | 1.94x10^-6^ |
|  | rs12589136 | 14 | 93863439 | T | G | 0.22 | 0.10 | 0.01 | 3.32x10^-12^ |
| Shin GWAS 2014 | rs1010874 | 10 | 84501091 | A | G | 0.06 | -0.05 | 0.01 | 3.17x10^-7^ |
|  | rs12883490 | 14 | 91195372 | T | C | 0.67 | 0.02 | 0.003 | 3.76x10^-7^ |
|  | rs1381274 | 14 | 98655131 | T | C | 0.47 | -0.01 | 0.003 | 3.63x10^-6^ |
|  | rs4439706 | 15 | 47129544 | T | C | 0.72 | 0.02 | 0.003 | 3.78x10^-6^ |
|  | rs11855136 | 15 | 57770091 | A | G | 0.04 | -0.06 | 0.01 | 2.99x10^-7^ |
| Long GWAS 2017 | rs4511131 | 1 | 101720655 | C | T | 0.13 | -0.24 | 0.06 | 3.96x10^-6^ |
|  | rs58891328 | 2 | 3825771 | T | C | 0.12 | -0.17 | 0.07 | 3.94x10^-6^ |
|  | rs2709379 | 2 | 208496182 | G | C | 0.32 | -0.13 | 0.05 | 1.09x10^-6^ |
|  | rs2366843 | 3 | 192314350 | T | C | 0.25 | 0.15 | 0.05 | 3.99x10^-6^ |
|  | rs140737699 | 4 | 31564620 | G | T | 0.01 | 1.21 | 0.27 | 2.14x10^-6^ |
|  | rs61258069 | 4 | 175600145 | C | T | 0.14 | 0.19 | 0.06 | 4.06x10^-6^ |
|  | rs59772690 | 5 | 38034250 | C | T | 0.04 | -0.49 | 0.12 | 1.11x10^-6^ |
|  | rs9328402 | 6 | 7227511 | T | C | 0.02 | 0.45 | 0.15 | 2.45x10^-6^ |
|  | rs7765517 | 6 | 33981782 | C | A | 0.02 | 0.62 | 0.15 | 3.87x10^-6^ |
|  | rs2721936^b^ | 8 | 116632819 | T | A | 0.57 | -0.19 | 0.04 | 2.87x10^-6^ |
|  | rs1962989 | 10 | 45074407 | T | C | 0.42 | 0.18 | 0.04 | 3.92x10^-6^ |
|  | rs56757634 | 10 | 116321830 | T | C | 0.04 | 0.39 | 0.11 | 4.65x10^-6^ |
|  | rs1860400 | 10 | 118072627 | T | C | 0.17 | -0.27 | 0.06 | 6.41x10^-7^ |
|  | rs11609525 | 12 | 22639669 | G | A | 0.13 | 0.23 | 0.06 | 6.65x10^-7^ |
|  | rs3783297 | 14 | 30064026 | C | T | 0.38 | -0.20 | 0.04 | 2.20x10^-6^ |
|  | rs62000804 | 14 | 44114760 | C | A | 0.04 | 0.43 | 0.11 | 3.77x10^-6^ |
|  | rs17810938 | 14 | 77480580 | C | A | 0.02 | 0.64 | 0.15 | 3.17x10^-6^ |
|  | rs117226077 | 19 | 29432034 | A | G | 0.03 | -0.52 | 0.12 | 2.13x10^-6^ |

Abbreviations: Chr, chromosome; EAF, effect allele frequency; SD, standard deviation; SNP, single nucleotide polymorphism.

^a^Source: SNP-exposure associations for cortisol were from

1) CORtisol NETwork (CORNET) consortium of 12,597 participants (mean age 53.5 years, 59.2% women) based on z-score of log-transformed plasma cortisol with estimates based on Crawford et al. *Eur J Endocrinol*. 2019;

2) GWAS of metabolomics by Shin et al. of 7,824 participants (mean age 55.1 years, 83.5% women) based on log-transformed plasma/serum cortisol; and

3) GWAS of metabolomics by Long et al. of 2,049 participants (median age 58.0 years, 96.6% women) based on log-transformed mean of median normalized value of serum cortisol from three visits.

^b^SNP (*rs2721936*) from Long GWAS with known horizontal pleiotropy (body size and composition, erythrocytes, leukocytes, and hematocrit) based on PhenoScanner.

Table S2. Association of genetically predicted cortisol (*P*-value<5x10^-6^ and r^2^<0.001) based on SNPs from the CORtisol NETwork (CORNET) consortium with socio-economic position (education, and Townsend deprivation index) and lifestyle (smoking, alcohol drinking, and physical activity) from the UK Biobank using Mendelian randomization (MR) with different methods

| Characteristics | SNPs | *F*-statistic | Method | Mean  difference | 95% CI |  | *P*-value | IVW | |  | MR-Egger | |
| --- | --- | --- | --- | --- | --- | --- | --- | --- | --- | --- | --- | --- |
|  |  |  |  |  |  |  |  | Cochran’s  *Q*-statistic | *P*-value |  | Intercept  *P*-value | I^2^ |
| Education | 6 | 28.3 | IVW | 0.006 | -0.010 | 0.021 | 0.49 | 2.14 | 0.83 |  |  |  |
|  |  |  | WM | 0.009 | -0.010 | 0.029 | 0.34 |  |  |  |  |  |
|  |  |  | MR-Egger | 0.015 | -0.011 | 0.040 | 0.26 |  |  |  | 0.37 | 71.3% |
|  |  |  | MR-PRESSO | 0.006 | -0.008 | 0.019 | 0.34 |  |  |  |  |  |
| Townsend | 6 | 28.3 | IVW | 0.006 | -0.010 | 0.022 | 0.47 | 1.41 | 0.92 |  |  |  |
| deprivation index |  |  | WM | 0.007 | -0.013 | 0.027 | 0.48 |  |  |  |  |  |
|  |  |  | MR-Egger | 0.011 | -0.015 | 0.037 | 0.41 |  |  |  | 0.64 | 71.5% |
|  |  |  | MR-PRESSO | 0.006 | -0.005 | 0.017 | 0.23 |  |  |  |  |  |
|  |  |  |  | Odds ratio | 95% CI |  | *P*-value | IVW | |  | MR-Egger | |
|  |  |  |  |  |  |  |  | Cochran’s  *Q*-statistic | *P*-value |  | Intercept  *P*-value | I^2^ |
| Alcohol drinking | 6 | 28.3 | IVW | 1.001 | 0.995 | 1.007 | 0.70 | 10.11 | 0.07 |  |  |  |
|  |  |  | WM | 0.998 | 0.993 | 1.003 | 0.43 |  |  |  |  |  |
|  |  |  | MR-Egger | 0.999 | 0.989 | 1.010 | 0.89 |  |  |  | 0.65 | 71.5% |
|  |  |  | MR-PRESSO | 1.001 | 0.994 | 1.009 | 0.72 |  |  |  |  |  |
| Smoking | 6 | 28.3 | IVW | 0.996 | 0.991 | 1.001 | 0.08 | 3.72 | 0.59 |  |  |  |
|  |  |  | WM | 0.995 | 0.989 | 1.002 | 0.17 |  |  |  | 0.96 | 71.5% |
|  |  |  | MR-Egger | 0.996 | 0.988 | 1.004 | 0.30 |  |  |  |  |  |
|  |  |  | MR-PRESSO | 0.996 | 0.990 | 1.001 | 0.10 |  |  |  |  |  |
| Physical activity | 6 | 28.3 | IVW | 0.997 | 0.949 | 1.047 | 0.90 | 7.88 | 0.16 |  |  |  |
|  |  |  | WM | 0.995 | 0.949 | 1.043 | 0.83 |  |  |  | 0.96 | 71.6% |
|  |  |  | MR-Egger | 0.995 | 0.911 | 1.087 | 0.91 |  |  |  |  |  |
|  |  |  | MR-PRESSO | 0.997 | 0.935 | 1.063 | 0.90 |  |  |  |  |  |

Abbreviations: CI, confidence interval; IVW, inverse variance weighting; MR, Mendelian randomization, SNP, single nucleotide polymorphism; WM, weighted median.

Table S3. Association of genetically predicted cortisol based on one single nucleotide polymorphism (SNP) reaching genome-wide significance (*P*-value<5x10^-6^ and r^2^<0.001) from Long GWAS after excluding one SNP with known horizontal pleiotropy (*rs2721936*) with ischemic heart disease (IHD) based on the CARDIoGRAMplusC4D 1000 Genomes-based GWAS (1000 Genomes) with replication based on the UK Biobank, ischemic stroke based on the MEGASTROKE and type 2 diabetes (T2DM) based on the DIAbetes Meta-ANalysis of Trans-Ethnic association studies (DIAMANTE) with checking based on the UK Biobank using Mendelian randomization (MR)^a^

| Exposure | Outcomes | SNPs | *F*-statistic | Method | Odds | 95% CI |  | *P*-value | IVW | |  | MR-Egger | |
| --- | --- | --- | --- | --- | --- | --- | --- | --- | --- | --- | --- | --- | --- |
| sources |  |  |  |  | ratio |  |  |  | Cochran’s  *Q*-statistic | *P*-value |  | Intercept  *P*-value | I^2^ |
| Long GWAS 2017 | 1000 Genomes | 17 | 14.7 | IVW | 1.02 | 0.99 | 1.05 | 0.27 | 20.84 | 0.18 |  |  |  |
|  |  |  |  | WM | 1.01 | 0.97 | 1.05 | 0.55 |  |  |  |  |  |
|  |  |  |  | MR-Egger | 1.03 | 0.96 | 1.09 | 0.43 |  |  |  | 0.76 | 0% |
|  |  |  |  | MR-PRESSO | 1.02 | 0.98 | 1.05 | 0.29 |  |  |  |  |  |
|  | UK Biobank | 17 | 14.7 | IVW | 0.99 | 0.97 | 1.02 | 0.61 | 12.11 | 0.74 |  |  |  |
|  |  |  |  | WM | 0.99 | 0.96 | 1.03 | 0.68 |  |  |  |  |  |
|  |  |  |  | MR-Egger | 0.99 | 0.94 | 1.05 | 0.73 |  |  |  | 0.91 | 0% |
|  |  |  |  | MR-PRESSO | 0.99 | 0.97 | 1.02 | 0.57 |  |  |  |  |  |
|  | MEGASTROKE | 17 | 14.7 | IVW | 1.00 | 0.97 | 1.03 | 0.91 | 8.93 | 0.92 |  |  |  |
|  |  |  |  | WM | 1.00 | 0.96 | 1.04 | 0.91 |  |  |  |  |  |
|  |  |  |  | MR-Egger | 1.02 | 0.96 | 1.09 | 0.46 |  |  |  | 0.43 | 0% |
|  |  |  |  | MR-PRESSO | 1.00 | 0.98 | 1.03 | 0.89 |  |  |  |  |  |
|  | DIAMANTE | 17 | 14.7 | IVW | 0.99 | 0.96 | 1.01 | 0.31 | 32.25 | 0.01 |  |  |  |
|  |  |  |  | WM | 0.97 | 0.94 | 1.00 | 0.03 |  |  |  |  |  |
|  |  |  |  | MR-Egger | 1.00 | 0.95 | 1.05 | 0.90 |  |  |  | 0.67 | 0% |
|  |  |  |  | MR-PRESSO | 0.99 | 0.96 | 1.01 | 0.33 |  |  |  |  |  |
|  | UK Biobank | 17 | 14.7 | IVW | 1.00 | 0.97 | 1.03 | 0.95 | 17.46 | 0.36 |  |  |  |
|  |  |  |  | WM | 1.00 | 0.96 | 1.05 | 0.99 |  |  |  |  |  |
|  |  |  |  | MR-Egger | 0.99 | 0.92 | 1.06 | 0.68 |  |  |  | 0.66 | 0% |
|  |  |  |  | MR-PRESSO | 1.00 | 0.96 | 1.03 | 0.95 |  |  |  |  |  |

Abbreviations: CI, confidence interval; IVW, inverse variance weighting; MR, Mendelian randomization, SNP, single nucleotide polymorphism; WM, weighted median.

Table S4. Association of genetically predicted cortisol based on one single nucleotide polymorphism (SNP) reaching genome-wide significance (*rs12589136*) (*P*-value<5x10^-8^ and r^2^<0.001) from CORtisol NETwork (CORNET) consortium with ischemic heart disease (IHD) based on the CARDIoGRAMplusC4D 1000 Genomes-based GWAS (1000 Genomes) with replication based on the UK Biobank, ischemic stroke based on the MEGASTROKE and type 2 diabetes (T2DM) based on the DIAbetes Meta-ANalysis of Trans-Ethnic association studies (DIAMANTE) with checking based on the UK Biobank using Mendelian randomization (MR)^a^

| Exposure | Outcome | SNP | *F*-statistic | Method | Odds | 95% CI |  | *P*-value |
| --- | --- | --- | --- | --- | --- | --- | --- | --- |
| sources | sources |  |  |  | ratio |  |  |  |
| CORNET 2014 | 1000 Genomes | 1 | 48.6 | Wald estimator | 1.00 | 0.82 | 1.23 | 0.99 |
|  |  |  |  |  |  |  |  |  |
|  | UK Biobank (IHD) | 1 | 48.6 | Wald estimator | 1.08 | 0.88 | 1.33 | 0.47 |
|  |  |  |  |  |  |  |  |  |
|  | MEGASTROKE | 1 | 48.6 | Wald estimator | 1.02 | 0.81 | 1.29 | 0.84 |
|  |  |  |  |  |  |  |  |  |
|  | DIAMANTE | 1 | 48.6 | Wald estimator | 1.08 | 0.93 | 1.25 | 0.33 |
|  |  |  |  |  |  |  |  |  |
|  | UK Biobank (T2DM) | 1 | 48.6 | Wald estimator | 0.79 | 0.61 | 1.02 | 0.07 |

Abbreviations: CI, confidence interval; MR, Mendelian randomization, SNP, single nucleotide polymorphism.

^a^Wald estimators were based on SNP on outcome divided by SNP on exposure, with standard error based on Fieller's Theorem.

Table S5. Association of genetically predicted cortisol based on 3 correlated SNPs used in Crawford et al. studies^a^ with ischemic heart disease based on the CARDIoGRAM (original dataset used in Crawford et al. 2015 poster abstract), CARDIoGRAMplusC4D 1000 Genomes-based GWAS (1000 Genomes), and a meta-analysis of UK Biobank and CARDIoGRAMplusC4D (CAD_META) (original dataset used in Crawford et al. 2019 paper) using Mendelian randomization (MR) with different methods

| Exposure | Outcome | SNPs | *F*-statistic | Method | Odds | 95% CI |  | *P*-value | IVW | |
| --- | --- | --- | --- | --- | --- | --- | --- | --- | --- | --- |
| sources | sources |  |  |  | ratio |  |  |  | Cochran’s  *Q*-statistic | *P*-value |
| Crawford 2019 | CARDIoGRAM | 3 | 40.6 | WGLR | 1.30 | 0.97 | 1.74 | 0.08 |  |  |
|  |  |  |  | IVW (correlated) | 1.30 | 0.95 | 1.78 | 0.10 | 2.34 | 0.31 |
|  |  |  |  |  |  |  |  |  |  |  |
|  | 1000 Genomes | 3 | 40.6 | WGLR | 1.08 | 0.91 | 1.29 | 0.39 |  |  |
|  |  |  |  | IVW (correlated) | 1.08 | 0.86 | 1.35 | 0.50 | 3.21 | 0.20 |
|  |  |  |  |  |  |  |  |  |  |  |
|  | CAD_META | 3 | 40.6 | WGLR | 1.06 | 0.95 | 1.18 | 0.29 |  |  |
|  |  |  |  | IVW (correlated) | 1.06 | 0.95 | 1.18 | 0.29 | 1.23 | 0.54 |

Abbreviations: CI, confidence interval; IVW, inverse variance weighting; MR, Mendelian randomization, SNP, single nucleotide polymorphism; WGLR, weighted generalized linear regression.

^a^References:

1. Crawford A, Timpson N, Davey Smith G, Walker B. Testing causality in the association of plasma cortisol with risk of coronary heart disease: a Mendelian randomisation study. *Heart*. 2015;101(Suppl 6):A6-A7; Poster was accessed via <https://www.endocrine-abstracts.org/ea/0038/eposters/ea0038P182_eposter.pdf>.

2. Crawford A, Soderberg S, Kirschbaum C, Murphy L, Eliasson M, Ebrahim S, et al. Morning plasma cortisol as a cardiovascular risk factor: findings from prospective cohort and Mendelian randomization studies. *Eur J Endocrinol*. 2019;pii: EJE-19-0161.R1.

Table S6. Association of genetically predicted cortisol based on 2 independent SNPs used in Pott et al. study^a^ with ischemic heart disease based on the CARDIoGRAMplusC4D 1000 Genomes-based GWAS (1000 Genomes) with replication based on the UK Biobank using Mendelian randomization (MR) with different methods

| Exposure | Outcome | SNPs | *F*-statistic | Method | Odds | 95% CI |  | *P*-value | IVW | |
| --- | --- | --- | --- | --- | --- | --- | --- | --- | --- | --- |
| sources | sources |  |  |  | ratio |  |  |  | Cochran’s  *Q*-statistic | *P*-value |
| Pott 2019 | 1000 Genomes | 2 | 33.3 | IVW | 0.84 | 0.54 | 1.30 | 0.43 | 1.39 | 0.24 |
|  |  |  |  |  |  |  |  |  |  |  |
|  | UK Biobank | 2 | 33.3 | IVW | 1.09 | 0.75 | 1.59 | 0.65 | 0.33 | 0.56 |

Abbreviations: CI, confidence interval; IVW, inverse variance weighting; MR, Mendelian randomization, SNP, single nucleotide polymorphism.

^a^Reference:

1. Pott J, Bae Y, Horn K, Teren A, Kuhnapfel A, Kirsten H, et al. Genetic association study of eight steroid hormones and implications for sexual dimorphism of coronary artery disease. *J Clin Endocrinol Metab*. 2019;pii: jc.2019-00757.
