# Supplementary material for: The role of cortisol in ischemic heart disease, ischemic stroke, type 2 diabetes, and cardiovascular disease risk factors: a bi-directional Mendelian randomization study
Source: BMC Med. 2020 Nov 27;18:363. doi: 10.1186/s12916-020-01831-3 (PMC7694946; doi:10.1186/s12916-020-01831-3)
Supplement: Supplementary file 2 — Additional file 2: Table S1. Single nucleotide polymorphisms (SNPs) considerably (P-value< 5 × 10^−6^) and independently (r2 < 0.001) associated with cortisol from three data sources (CORtisol NETwork (CORNET) consortium, Shin GWAS and Long GWAS) using P-value based effect size with sample overlap correctiona (total SNPs = 29). Table S2. Association of genetically predicted cortisol (P-value< 5 × 10^−6^ and r2 < 0.001) based on single nucleotide polymorphisms (SNPs) from three data sources (CORtisol NETwork (CORNET) consortium, Shin GWAS and Long GWAS) using p-value based effect size with sample overlap correction with ischemic heart disease (IHD) based on the CARDIoGRAMplusC4D 1000 Genomes-based GWAS (1000 Genomes) with replication based on the UK Biobank using Mendelian randomization (MR) with different methods. Table S3. Association of genetically predicted cortisol (P-value< 5 × 10^−6^ and r2 < 0.001) based on single nucleotide polymorphisms (SNPs) from three data sources (CORtisol NETwork (CORNET) consortium, Shin GWAS and Long GWAS) using p-value based effect size with sample overlap correction with ischemic stroke based on the MEGASTROKE using Mendelian randomization (MR) with different methods. Table S4. Association of genetically predicted cortisol (P-value< 5 × 10^−6^ and r2 < 0.001) based on single nucleotide polymorphisms (SNPs) from three data sources (CORtisol NETwork (CORNET) consortium, Shin GWAS and Long GWAS) using p-value based effect size with sample overlap correction with type 2 diabetes (T2DM) based on the DIAbetes Meta-ANalysis of Trans-Ethnic association studies (DIAMANTE) with checking based on the UK Biobank using Mendelian randomization (MR) with different methods. [file 12916_2020_1831_MOESM2_ESM.docx]

**Additional file 2**

Table S1. Single nucleotide polymorphisms (SNPs) considerably (*P*-value<5x10^-6^) and independently (r^2^<0.001) associated with cortisol from three data sources (CORtisol NETwork (CORNET) consortium, Shin GWAS and Long GWAS) using p-value based effect size with sample overlap correction^a^ (total SNPs=29)

| Source | SNP | Chr | Position | Effect  allele | Other  allele | EAF | Sample size | Effect size | SD | *P*-value |
| --- | --- | --- | --- | --- | --- | --- | --- | --- | --- | --- |
| CORNET 2014 | rs1340395 | 1 | 102662715 | T | C | 0.93 | 12592 | -0.13 | 0.03 | 1.09x10^-6^ |
|  | rs17029942 | 3 | 3320289 | G | A | 0.97 | 1190 | -0.66 | 0.13 | 3.10x10^-7^ |
|  | rs4400057 | 4 | 58712526 | A | G | 0.91 | 2070 | -0.32 | 0.07 | 9.46x10^-7^ |
|  | rs1075533 | 11 | 102963776 | G | A | 0.96 | 12589 | 0.17 | 0.03 | 7.74x10^-7^ |
|  | rs6830 | 14 | 73238184 | G | A | 0.68 | 12589 | 0.06 | 0.01 | 1.94x10^-6^ |
|  | rs12589136 | 14 | 93863439 | T | G | 0.22 | 12589 | 0.10 | 0.01 | 3.32x10^-12^ |
| Shin GWAS 2014 | rs1010874 | 10 | 84501091 | A | G | 0.06 | 7795 | -0.05 | 0.01 | 3.17x10^-7^ |
|  | rs12883490 | 14 | 91195372 | T | C | 0.67 | 7795 | 0.02 | 0.003 | 3.76x10^-7^ |
|  | rs1381274 | 14 | 98655131 | T | C | 0.47 | 7795 | -0.02 | 0.003 | 3.63x10^-6^ |
|  | rs4439706 | 15 | 47129544 | T | C | 0.72 | 7795 | 0.02 | 0.003 | 3.78x10^-6^ |
|  | rs11855136 | 15 | 57770091 | A | G | 0.04 | 7795 | -0.06 | 0.01 | 2.99x10^-7^ |
| Long GWAS 2017 | rs4511131 | 1 | 101720655 | C | T | 0.13 | 1957 | -0.31 | 0.06 | 3.96x10^-6^ |
|  | rs58891328 | 2 | 3825771 | T | C | 0.12 | 1957 | -0.32 | 0.07 | 3.94x10^-6^ |
|  | rs2709379 | 2 | 208496182 | G | C | 0.32 | 1957 | -0.23 | 0.05 | 1.09x10^-6^ |
|  | rs2366843 | 3 | 192314350 | T | C | 0.25 | 1957 | 0.23 | 0.05 | 3.99x10^-6^ |
|  | rs140737699 | 4 | 31564620 | G | T | 0.01 | 1957 | 1.30 | 0.27 | 2.14x10^-6^ |
|  | rs61258069 | 4 | 175600145 | C | T | 0.14 | 1957 | 0.29 | 0.06 | 4.06x10^-6^ |
|  | rs59772690 | 5 | 38034250 | C | T | 0.04 | 1957 | -0.60 | 0.12 | 1.11x10^-6^ |
|  | rs9328402 | 6 | 7227511 | T | C | 0.02 | 1957 | 0.74 | 0.15 | 2.45x10^-6^ |
|  | rs7765517 | 6 | 33981782 | C | A | 0.02 | 1957 | 0.73 | 0.15 | 3.87x10^-6^ |
|  | rs2721936^b^ | 8 | 116632819 | T | A | 0.57 | 1957 | -0.21 | 0.04 | 2.87x10^-6^ |
|  | rs1962989 | 10 | 45074407 | T | C | 0.42 | 1957 | 0.20 | 0.04 | 3.92x10^-6^ |
|  | rs56757634 | 10 | 116321830 | T | C | 0.04 | 1957 | 0.51 | 0.11 | 4.65x10^-6^ |
|  | rs1860400 | 10 | 118072627 | T | C | 0.17 | 1957 | -0.29 | 0.06 | 6.41x10^-7^ |
|  | rs11609525 | 12 | 22639669 | G | A | 0.13 | 1957 | 0.33 | 0.06 | 6.65x10^-7^ |
|  | rs3783297 | 14 | 30064026 | C | T | 0.38 | 1957 | -0.22 | 0.04 | 2.20x10^-6^ |
|  | rs62000804 | 14 | 44114760 | C | A | 0.04 | 1957 | 0.54 | 0.11 | 3.77x10^-6^ |
|  | rs17810938 | 14 | 77480580 | C | A | 0.02 | 1957 | 0.71 | 0.15 | 3.17x10^-6^ |
|  | rs117226077 | 19 | 29432034 | A | G | 0.03 | 1957 | -0.58 | 0.12 | 2.13x10^-6^ |

Abbreviations: Chr, chromosome; EAF, effect allele frequency; SD, standard deviation; SNP, single nucleotide polymorphism.

^a^Effect size for each SNP was derived from p-value based on sample size with correction for sample overlap given one-third of sample in Long GWAS 2017 was also included in Shin GWAS 2014.

Table S2. Association of genetically predicted cortisol (*P*-value<5x10^-6^ and r^2^<0.001) based on single nucleotide polymorphisms (SNPs) from three data sources (CORtisol NETwork (CORNET) consortium, Shin GWAS and Long GWAS) using p-value based effect size with sample overlap correction with ischemic heart disease (IHD) based on the CARDIoGRAMplusC4D 1000 Genomes-based GWAS (1000 Genomes) with replication based on the UK Biobank using Mendelian randomization (MR) with different methods

| Exposure | Outcome | SNPs | *F-statistic* | Method | Odds | 95% CI |  | *P-value* | IVW |  |  | MR-Egger | |
| --- | --- | --- | --- | --- | --- | --- | --- | --- | --- | --- | --- | --- | --- |
| sources | sources |  |  |  | ratio |  |  |  | Cochran’s  *Q*-statistic | *P*-value |  | Intercept  *P*-value | I^2^ |
| All 3 GWAS | 1000 Genomes | 29 | 24.9 | IVW | 1.01 | 0.99 | 1.03 | 0.34 | 31.90 | 0.28 |  |  |  |
|  |  |  |  | WM | 1.00 | 0.98 | 1.03 | 0.72 |  |  |  |  |  |
|  |  |  |  | MR-Egger | 1.02 | 0.99 | 1.06 | 0.14 |  |  |  | 0.26 | 93.7% |
|  |  |  |  | MR-PRESSO | 1.01 | 0.99 | 1.03 | 0.35 |  |  |  |  |  |
|  | UK Biobank | 29 | 24.9 | IVW | 0.99 | 0.97 | 1.01 | 0.33 | 24.13 | 0.67 |  |  |  |
|  |  |  |  | WM | 0.99 | 0.97 | 1.02 | 0.63 |  |  |  |  |  |
|  |  |  |  | MR-Egger | 1.00 | 0.97 | 1.03 | 0.86 |  |  |  | 0.32 | 93.7% |
|  |  |  |  | MR-PRESSO | 0.99 | 0.97 | 1.01 | 0.31 |  |  |  |  |  |

Abbreviations: CI, confidence interval; IVW, inverse variance weighting; MR, Mendelian randomization, SNP, single nucleotide polymorphism; WM, weighted median.

Table S3. Association of genetically predicted cortisol (*P*-value<5x10^-6^ and r^2^<0.001) based on single nucleotide polymorphisms (SNPs) from three data sources (CORtisol NETwork (CORNET) consortium, Shin GWAS and Long GWAS) using p-value based effect size with sample overlap correction with ischemic stroke based on the MEGASTROKE using Mendelian randomization (MR) with different methods

| Exposure | Outcome | SNPs | *F*-statistic | Method | Odds | 95% CI |  | *P*-value | IVW | |  | MR-Egger | |
| --- | --- | --- | --- | --- | --- | --- | --- | --- | --- | --- | --- | --- | --- |
| sources | sources |  |  |  | ratio |  |  |  | Cochran’s  *Q*-statistic | *P*-value |  | Intercept  *P*-value | I^2^ |
| All 3 GWAS | MEGASTROKE | 29 | 24.9 | IVW | 1.00 | 0.97 | 1.02 | 0.88 | 34.20 | 0.19 |  |  |  |
|  |  |  |  | WM | 1.00 | 0.97 | 1.03 | 0.89 |  |  |  |  |  |
|  |  |  |  | MR-Egger | 1.04 | 1.00 | 1.07 | 0.05 |  |  |  | 0.01 | 93.7% |
|  |  |  |  | MR-PRESSO | 1.00 | 0.97 | 1.02 | 0.88 |  |  |  |  |  |

Abbreviations: CI, confidence interval; IVW, inverse variance weighting; MR, Mendelian randomization, SNP, single nucleotide polymorphism; WM, weighted median.

Table S4. Association of genetically predicted cortisol (*P*-value<5x10^-6^ and r^2^<0.001) based on single nucleotide polymorphisms (SNPs) from three data sources (CORtisol NETwork (CORNET) consortium, Shin GWAS and Long GWAS) using p-value based effect size with sample overlap correction with type 2 diabetes (T2DM) based on the DIAbetes Meta-ANalysis of Trans-Ethnic association studies (DIAMANTE) with checking based on the UK Biobank using Mendelian randomization (MR) with different methods

| Exposure | Outcome | SNPs | *F*-statistic | Method | Odds | 95% CI |  | *P*-value | IVW | |  | MR-Egger | |
| --- | --- | --- | --- | --- | --- | --- | --- | --- | --- | --- | --- | --- | --- |
| sources | sources |  |  |  | ratio |  |  |  | Cochran’s  *Q*-statistic | *P*-value |  | Intercept  *P*-value | I^2^ |
| All 3 GWAS | DIAMANTE | 29 | 24.9 | IVW | 1.00 | 0.97 | 1.02 | 0.86 | 74.77 | <0.001 |  |  |  |
|  |  |  |  | WM | 0.98 | 0.96 | 1.01 | 0.19 |  |  |  |  |  |
|  |  |  |  | MR-Egger | 1.01 | 0.97 | 1.05 | 0.79 |  |  |  | 0.62 | 91.0% |
|  |  |  |  | MR-PRESSO^a^ | 0.99 | 0.96 | 1.01 | 0.28 |  |  |  |  |  |
|  | UK Biobank | 29 | 24.9 | IVW | 1.01 | 0.97 | 1.04 | 0.64 | 40.46 | 0.06 |  |  |  |
|  |  |  |  | WM | 1.01 | 0.97 | 1.04 | 0.61 |  |  |  |  |  |
|  |  |  |  | MR-Egger | 1.03 | 0.98 | 1.08 | 0.21 |  |  |  | 0.22 | 93.7% |
|  |  |  |  | MR-PRESSO | 1.01 | 0.98 | 1.04 | 0.64 |  |  |  |  |  |

Abbreviations: CI, confidence interval; IVW, inverse variance weighting; MR, Mendelian randomization, SNP, single nucleotide polymorphism; WM, weighted median.

^a^MR-PRESSO estimate was obtained by excluding 1 outlier (*rs2721936*).
