# Supplementary material for: The role of cortisol in ischemic heart disease, ischemic stroke, type 2 diabetes, and cardiovascular disease risk factors: a bi-directional Mendelian randomization study
Source: BMC Med. 2020 Nov 27;18:363. doi: 10.1186/s12916-020-01831-3 (PMC7694946; doi:10.1186/s12916-020-01831-3)
Supplement: Supplementary file 3 — Additional file 3: Table S1. Single nucleotide polymorphisms (SNPs) considerably (P-value< 5 × 10^−6^) and independently (r2 < 0.001) associated with cortisol from three data sources (CORtisol NETwork (CORNET) consortium, Shin GWAS and Long GWAS) using estimates based on Crawford et al. Eur J Endocrinol. 2019a (total SNPs = 23). Table S2. Association of genetically predicted cortisol (P-value< 5 × 10^−6^ and r2 < 0.001) based on single nucleotide polymorphisms (SNPs) from three data sources (CORtisol NETwork (CORNET) consortium, Shin GWAS and Long GWAS) using estimates based on Crawford et al. [19] study with ischemic heart disease (IHD) based on the CARDIoGRAMplusC4D 1000 Genomes-based GWAS (1000 Genomes) with replication based on the UK Biobank using Mendelian randomization (MR) with different methods. Table S3. Association of genetically predicted cortisol (P-value< 5 × 10^−6^ and r2 < 0.001) based on single nucleotide polymorphisms (SNPs) from three data sources (CORtisol NETwork (CORNET) consortium, Shin GWAS and Long GWAS) using estimates based on Crawford et al. [19] study with ischemic stroke based on the MEGASTROKE using Mendelian randomization (MR) with different methods. Table S4. Association of genetically predicted cortisol (P-value< 5 × 10^−6^ and r2 < 0.001) based on single nucleotide polymorphisms (SNPs) from three data sources (CORtisol NETwork (CORNET) consortium, Shin GWAS and Long GWAS) using estimates based on Crawford et al. [19] study with type 2 diabetes (T2DM) based on the DIAbetes Meta-ANalysis of Trans-Ethnic association studies (DIAMANTE) with checking based on the UK Biobank using Mendelian randomization (MR) with different methods. [file 12916_2020_1831_MOESM3_ESM.docx]

**Additional file 3**

Table S1. Single nucleotide polymorphisms (SNPs) considerably (*P*-value<5x10^-6^) and independently (r^2^<0.001) associated with cortisol from three data sources (CORtisol NETwork (CORNET) consortium, Shin GWAS and Long GWAS) using estimates based on Crawford et al. Eur J Endocrinol. 2019^a^ (total SNPs=23)

| Source | Original  SNP | Proxy  SNP^b^ | Chr | Position | Effect  allele | Other  allele | EAF | Beta | SD | *P*-value |
| --- | --- | --- | --- | --- | --- | --- | --- | --- | --- | --- |
| CORNET 2014 | rs1340395 |  | 1 | 102662715 | T | C | 0.93 | -0.13 | 0.03 | 1.09x10^-6^ |
|  | rs17029942 |  | 3 | 3320289 | G | A | 0.97 | -0.65 | 0.13 | 3.10x10^-7^ |
|  | rs4400057 |  | 4 | 58712526 | A | G | 0.91 | -0.32 | 0.07 | 9.46x10^-7^ |
|  | rs1075533 |  | 11 | 102963776 | G | A | 0.96 | 0.17 | 0.03 | 7.74x10^-7^ |
|  | rs6830 |  | 14 | 73238184 | G | A | 0.68 | 0.06 | 0.01 | 1.94x10^-6^ |
|  | rs12589136 |  | 14 | 93863439 | T | G | 0.22 | 0.10 | 0.01 | 3.32x10^-12^ |
| Shin GWAS 2014 | rs1010874 |  | 10 | 84501091 | A | G | 0.95 | 0.01 | 0.07 | 0.86 |
|  | rs12883490 |  | 14 | 91195372 | T | C | 0.65 | -0.004 | 0.01 | 0.79 |
|  | rs1381274 |  | 14 | 98655131 | T | C | 0.49 | -0.01 | 0.01 | 0.68 |
|  | rs4439706 |  | 15 | 47129544 | T | C | 0.72 | 0.01 | 0.01 | 0.34 |
|  | rs11855136 |  | 15 | 57770091 | A | G | 0.97 | -0.0004 | 0.07 | 0.995 |
| Long GWAS 2017 | rs4511131 | rs4511131 | 1 | 101720655 | C | T | 0.13 | NA |  |  |
|  | rs58891328 | rs17018001 | 2 | 3824123 | T | C | 0.11 | 0.0004 | 0.02 | 0.98 |
|  | rs2709379 | rs2551941 | 2 | 208492143 | G | C | 0.35 | -0.004 | 0.01 | 0.79 |
|  | rs2366843 | rs1827545 | 3 | 192314526 | T | C | 0.29 | 0.01 | 0.01 | 0.38 |
|  | rs140737699 | rs140737699 | 4 | 31564620 | G | T | 0.01 | NA |  |  |
|  | rs61258069 | rs1022789 | 4 | 175596896 | C | T | 0.89 | -0.004 | 0.02 | 0.83 |
|  | rs59772690 | rs1355092 | 5 | 38034726 | C | T | 0.03 | 0.004 | 0.04 | 0.92 |
|  | rs9328402 | rs6907334 | 6 | 7228816 | T | C | 0.97 | 0.03 | 0.06 | 0.54 |
|  | rs7765517 | rs9461939 | 6 | 33982796 | C | A | 0.03 | 0.03 | 0.05 | 0.53 |
|  | rs2721936 |  | 8 | 116632819 | T | A | 0.58 | -0.03 | 0.01 | 0.03 |
|  | rs1962989 | rs122244967 | 10 | 45077687 | T | C | 0.47 | -0.02 | 0.01 | 0.22 |
|  | rs56757634 | rs56757634 | 10 | 116321830 | T | C | 0.04 | NA |  |  |
|  | rs1860400 |  | 10 | 118072627 | T | C | 0.18 | -0.02 | 0.02 | 0.19 |
|  | rs11609525 |  | 12 | 22639669 | G | A | 0.12 | 0.01 | 0.02 | 0.58 |
|  | rs3783297 |  | 14 | 30064026 | C | T | 0.65 | 0.01 | 0.01 | 0.38 |
|  | rs62000804 | rs62000804 | 14 | 44114760 | C | A | 0.04 | NA |  |  |
|  | rs17810938 | rs17810938 | 14 | 77480580 | C | A | 0.02 | NA |  |  |
|  | rs117226077 | rs117226077 | 19 | 29432034 | A | G | 0.03 | NA |  |  |

Abbreviations: Chr, chromosome; EAF, effect allele frequency; NA, not applicable; SD, standard deviation; SNP, single nucleotide polymorphism.

^a^Source: SNP-exposure associations for cortisol (z-score of log-transformed plasma cortisol) for all three sources were from estimates based on Crawford et al. *Eur J Endocrinol*. 2019.

^b^Proxy SNPs (r^2^>0.8) in Europeans obtained from LDLink was used for any SNP unavailable based on Crawford et al. study.

^a^References:

1. Crawford A, Soderberg S, Kirschbaum C, Murphy L, Eliasson M, Ebrahim S, et al. Morning plasma cortisol as a cardiovascular risk factor: findings from prospective cohort and Mendelian randomization studies. *Eur J Endocrinol*. 2019;pii: EJE-19-0161.R1.

Table S2. Association of genetically predicted cortisol (*P*-value<5x10^-6^ and r^2^<0.001) based on single nucleotide polymorphisms (SNPs) from three data sources (CORtisol NETwork (CORNET) consortium, Shin GWAS and Long GWAS) using estimates based on Crawford et al. 2019 study with ischemic heart disease (IHD) based on the CARDIoGRAMplusC4D 1000 Genomes-based GWAS (1000 Genomes) with replication based on the UK Biobank using Mendelian randomization (MR) with different methods

| Exposure | Outcome | SNPs | *F-statistic* | Method | Odds | 95% CI |  | *P-value* | IVW |  |  | MR-Egger | |
| --- | --- | --- | --- | --- | --- | --- | --- | --- | --- | --- | --- | --- | --- |
| sources | sources |  |  |  | ratio |  |  |  | Cochran’s  *Q*-statistic | *P*-value |  | Intercept  *P*-value | I^2^ |
| Crawford 2019 | 1000 Genomes | 23 | 7.9 | IVW | 0.98 | 0.93 | 1.03 | 0.36 | 20.85 | 0.53 |  |  |  |
|  |  |  |  | WM | 1.00 | 0.93 | 1.07 | 0.95 |  |  |  |  |  |
|  |  |  |  | MR-Egger | 0.99 | 0.93 | 1.05 | 0.76 |  |  |  | 0.23 | 81.4% |
|  |  |  |  | MR-PRESSO | 0.98 | 0.93 | 1.03 | 0.35 |  |  |  |  |  |
|  | UK Biobank | 23 | 7.9 | IVW | 0.98 | 0.92 | 1.04 | 0.50 | 21.47 | 0.49 |  |  |  |
|  |  |  |  | WM | 0.99 | 0.92 | 1.07 | 0.82 |  |  |  |  |  |
|  |  |  |  | MR-Egger | 1.01 | 0.95 | 1.09 | 0.68 |  |  |  | 0.03 | 80.5% |
|  |  |  |  | MR-PRESSO | 0.98 | 0.92 | 1.04 | 0.50 |  |  |  |  |  |

Abbreviations: CI, confidence interval; IVW, inverse variance weighting; MR, Mendelian randomization, SNP, single nucleotide polymorphism; WM, weighted median.

Table S3. Association of genetically predicted cortisol (*P*-value<5x10^-6^ and r^2^<0.001) based on single nucleotide polymorphisms (SNPs) from three data sources (CORtisol NETwork (CORNET) consortium, Shin GWAS and Long GWAS) using estimates based on Crawford et al. 2019 study with ischemic stroke based on the MEGASTROKE using Mendelian randomization (MR) with different methods

| Exposure | Outcome | SNPs | *F*-statistic | Method | Odds | 95% CI |  | *P*-value | IVW | |  | MR-Egger | |
| --- | --- | --- | --- | --- | --- | --- | --- | --- | --- | --- | --- | --- | --- |
| sources | sources |  |  |  | ratio |  |  |  | Cochran’s  *Q*-statistic | *P*-value |  | Intercept  *P*-value | I^2^ |
| Crawford 2019 | MEGASTROKE | 23 | 7.9 | IVW | 0.99 | 0.91 | 1.07 | 0.71 | 30.23 | 0.11 |  |  |  |
|  |  |  |  | WM | 0.99 | 0.91 | 1.08 | 0.84 |  |  |  |  |  |
|  |  |  |  | MR-Egger | 1.00 | 0.92 | 1.10 | 0.98 |  |  |  | 0.45 | 80.7% |
|  |  |  |  | MR-PRESSO | 0.99 | 0.91 | 1.07 | 0.71 |  |  |  |  |  |

Abbreviations: CI, confidence interval; IVW, inverse variance weighting; MR, Mendelian randomization, SNP, single nucleotide polymorphism; WM, weighted median.

Table S4. Association of genetically predicted cortisol (*P*-value<5x10^-6^ and r^2^<0.001) based on single nucleotide polymorphisms (SNPs) from three data sources (CORtisol NETwork (CORNET) consortium, Shin GWAS and Long GWAS) using estimates based on Crawford et al. 2019 study with type 2 diabetes (T2DM) based on the DIAbetes Meta-ANalysis of Trans-Ethnic association studies (DIAMANTE) with checking based on the UK Biobank using Mendelian randomization (MR) with different methods

| Exposure | Outcome | SNPs | *F*-statistic | Method | Odds | 95% CI |  | *P*-value | IVW | |  | MR-Egger | |
| --- | --- | --- | --- | --- | --- | --- | --- | --- | --- | --- | --- | --- | --- |
| sources | sources |  |  |  | ratio |  |  |  | Cochran’s  *Q*-statistic | *P*-value |  | Intercept  *P*-value | I^2^ |
| Crawford 2019 | DIAMANTE | 23 | 7.9 | IVW | 1.004 | 0.94 | 1.08 | 0.92 | 59.18 | <0.001 |  |  |  |
|  |  |  |  | WM | 0.99 | 0.93 | 1.06 | 0.83 |  |  |  |  |  |
|  |  |  |  | MR-Egger | 1.00 | 0.92 | 1.09 | 0.97 |  |  |  | 0.92 | 80.8% |
|  |  |  |  | MR-PRESSO^a^ | 0.99 | 0.94 | 1.05 | 0.83 |  |  |  |  |  |
|  | UK Biobank | 23 | 7.9 | IVW | 1.01 | 0.91 | 1.11 | 0.88 | 36.41 | 0.03 |  |  |  |
|  |  |  |  | WM | 1.02 | 0.92 | 1.13 | 0.68 |  |  |  |  |  |
|  |  |  |  | MR-Egger | 1.03 | 0.92 | 1.15 | 0.58 |  |  |  | 0.36 | 80.6% |
|  |  |  |  | MR-PRESSO | 1.01 | 0.91 | 1.11 | 0.88 |  |  |  |  |  |

Abbreviations: CI, confidence interval; IVW, inverse variance weighting; MR, Mendelian randomization, SNP, single nucleotide polymorphism; WM, weighted median.

^a^MR-PRESSO estimate was obtained by excluding 1 outlier (*rs2721936*).
