# Supplementary material for: The role of cortisol in ischemic heart disease, ischemic stroke, type 2 diabetes, and cardiovascular disease risk factors: a bi-directional Mendelian randomization study
Source: BMC Med. 2020 Nov 27;18:363. doi: 10.1186/s12916-020-01831-3 (PMC7694946; doi:10.1186/s12916-020-01831-3)
Supplement: Supplementary file 4 — Additional file 4: Table S1. Single nucleotide polymorphisms (SNPs) considerably (P-value< 5 × 10^−6^) and independently (r2 < 0.001) associated with cortisol from a meta-analysis across all SNPs available in three data sources (CORtisol NETwork (CORNET) consortium, Shin GWAS and Long GWAS) using P-value based effect size with sample overlap correctiona (total SNPs = 42). Table S2. Association of genetically predicted cortisol (P-value< 5 × 10^−6^ and r2 < 0.001) based on single nucleotide polymorphisms (SNPs) from a meta-analysis across all SNPs available in three data sources (CORtisol NETwork (CORNET) consortium, Shin GWAS and Long GWAS) using p-value based effect size with sample overlap correction with ischemic heart disease (IHD) based on the CARDIoGRAMplusC4D 1000 Genomes-based GWAS (1000 Genomes) with replication based on the UK Biobank using Mendelian randomization (MR) with different methods. Table S3. Association of genetically predicted cortisol (P-value< 5 × 10^−6^ and r2 < 0.001) based on single nucleotide polymorphisms (SNPs) from a meta-analysis across all SNPs available in three data sources (CORtisol NETwork (CORNET) consortium, Shin GWAS and Long GWAS) using p-value based effect size with sample overlap correction with ischemic stroke based on the MEGASTROKE using Mendelian randomization (MR) with different methods. Table S4. Association of genetically predicted cortisol (P-value< 5 × 10^−6^ and r2 < 0.001) based on single nucleotide polymorphisms (SNPs) from a meta-analysis across all SNPs available in three data sources (CORtisol NETwork (CORNET) consortium, Shin GWAS and Long GWAS) using p-value based effect size with sample overlap correction with type 2 diabetes (T2DM) based on the DIAbetes Meta-ANalysis of Trans-Ethnic association studies (DIAMANTE) with checking based on the UK Biobank using Mendelian randomization (MR) with different methods. [file 12916_2020_1831_MOESM4_ESM.docx]

**Additional file 4**

Table S1. Single nucleotide polymorphisms (SNPs) considerably (*P*-value<5x10^-6^) and independently (r^2^<0.001) associated with cortisol from a meta-analysis across all SNPs available in three data sources (CORtisol NETwork (CORNET) consortium, Shin GWAS and Long GWAS) using p-value based effect size with sample overlap correction^a^ (total SNPs=42)

| Source | Original  SNP | Chr | Position | Effect  allele | Other  allele | EAF | Beta | SD | *P*-value |
| --- | --- | --- | --- | --- | --- | --- | --- | --- | --- |
| Meta-analysis | rs1340395 | 1 | 102662715 | T | C | 0.93 | -0.13 | 0.03 | 1.08x10^-6^ |
|  | rs4511131 | 1 | 101255099 | T | C | 0.87 | 0.31 | 0.06 | 1.98x10^-6^ |
|  | rs114129389 | 2 | 227747828 | A | G | 0.08 | 0.37 | 0.08 | 4.99x10^-6^ |
|  | rs2709379 | 2 | 207631458 | C | G | 0.68 | 0.23 | 0.05 | 5.46x10^-7^ |
|  | rs58891328 | 2 | 3778181 | T | C | 0.12 | -0.32 | 0.07 | 1.97x10^-6^ |
|  | rs80176033 | 2 | 20100334 | T | C | 0.98 | 0.72 | 0.15 | 2.81x10^-6^ |
|  | rs17029942 | 3 | 3320289 | A | G | 0.03 | 0.66 | 0.13 | 2.65x10^-7^ |
|  | rs2366843 | 3 | 192596561 | T | C | 0.25 | 0.23 | 0.05 | 1.99x10^-6^ |
|  | rs342924 | 3 | 146573712 | A | C | 0.31 | 0.22 | 0.05 | 2.51x10^-6^ |
|  | rs9653917 | 3 | 119130451 | A | G | 0.58 | 0.20 | 0.04 | 3.68x10^-6^ |
|  | rs140737699 | 4 | 31562998 | T | G | 0.99 | -1.30 | 0.27 | 1.07x10^-6^ |
|  | rs4400057 | 4 | 58712526 | A | G | 0.91 | -0.32 | 0.07 | 8.77x10^-7^ |
|  | rs61258069 | 4 | 174678994 | T | C | 0.86 | -0.29 | 0.06 | 2.03x10^-6^ |
|  | rs112809579 | 5 | 16768809 | A | G | 0.20 | -0.25 | 0.05 | 4.76x10^-6^ |
|  | rs59772690 | 5 | 38034148 | T | C | 0.96 | 0.60 | 0.12 | 5.56x10^-7^ |
|  | rs12210538 | 6 | 110866701 | A | G | 0.84 | 0.03 | 0.01 | 1.97x10^-6^ |
|  | rs7765517 | 6 | 34014005 | A | C | 0.98 | -0.73 | 0.15 | 1.94x10^-6^ |
|  | rs9328402 | 6 | 7227278 | T | C | 0.02 | 0.74 | 0.15 | 1.22x10^-6^ |
|  | rs3800637 | 7 | 137053972 | T | C | 0.67 | -0.01 | 0.00 | 3.92x10^-6^ |
|  | rs73196992 | 7 | 113045561 | T | G | 0.02 | -0.79 | 0.17 | 3.66x10^-6^ |
|  | rs9647693 | 7 | 158152993 | A | G | 0.29 | -0.26 | 0.06 | 2.89x10^-6^ |
|  | rs1448767 | 8 | 29846448 | T | C | 0.63 | -0.01 | 0.00 | 3.99x10^-6^ |
|  | rs4326410 | 8 | 115581355 | T | C | 0.57 | -0.21 | 0.04 | 1.89x10^-6^ |
|  | rs146672796 | 9 | 118223606 | A | C | 0.03 | -0.61 | 0.13 | 4.25x10^-6^ |
|  | rs1010874 | 10 | 84491071 | A | G | 0.06 | -0.05 | 0.01 | 1.80x10^-7^ |
|  | rs1962989 | 10 | 44578959 | T | C | 0.42 | 0.20 | 0.04 | 1.96x10^-6^ |
|  | rs56757634 | 10 | 114562071 | T | C | 0.04 | 0.51 | 0.11 | 2.33x10^-6^ |
|  | rs61864760 | 10 | 116257875 | A | C | 0.80 | 0.27 | 0.05 | 6.32x10^-7^ |
|  | rs1075533 | 11 | 102963776 | A | G | 0.04 | -0.17 | 0.03 | 7.64x10^-7^ |
|  | rs219918 | 12 | 13991396 | C | G | 0.02 | -0.05 | 0.01 | 2.13x10^-6^ |
|  | rs35607914 | 12 | 22410918 | A | G | 0.12 | 0.30 | 0.07 | 2.72x10^-6^ |
|  | rs4145213 | 13 | 108298270 | T | G | 0.60 | 0.18 | 0.04 | 3.90x10^-6^ |
|  | rs494147 | 13 | 111945705 | T | C | 0.46 | 0.20 | 0.04 | 3.05x10^-6^ |
|  | rs17810938 | 14 | 77014237 | A | C | 0.98 | -0.71 | 0.15 | 1.59x10^-6^ |

Table S1.

| Source | Original  SNP | Chr | Position | Effect  allele | Other  allele | EAF | Beta | SD | *P*-value |
| --- | --- | --- | --- | --- | --- | --- | --- | --- | --- |
| Meta-analysis | rs2749527 | 14 | 93896821 | T | C | 0.49 | -0.08 | 0.01 | 5.01x10^-11^ |
|  | rs62000804 | 14 | 43645557 | A | C | 0.96 | -0.54 | 0.11 | 1.88x10^-6^ |
|  | rs6830 | 14 | 73238184 | A | G | 0.32 | -0.06 | 0.01 | 1.92x10^-6^ |
|  | rs11855136 | 15 | 55557383 | A | G | 0.04 | -0.06 | 0.01 | 2.34x10^-7^ |
|  | rs304949 | 15 | 67962776 | T | C | 0.14 | -0.02 | 0.00 | 3.62x10^-6^ |
|  | rs4439706 | 15 | 44916836 | T | C | 0.72 | 0.02 | 0.00 | 1.20x10^-6^ |
|  | rs72720022 | 15 | 44727061 | T | C | 0.93 | -0.39 | 0.08 | 3.00x10^-6^ |
|  | rs117226077 | 19 | 28941127 | A | G | 0.03 | -0.58 | 0.12 | 1.06x10^-6^ |

Abbreviations: Chr, chromosome; EAF, effect allele frequency; SD, standard deviation; SNP, single nucleotide polymorphism.

^a^Effect size for each SNP was derived from p-value based on sample size with correction for sample overlap given one-third of sample in Long GWAS 2017 was also included in Shin GWAS 2014.

Table S2. Association of genetically predicted cortisol (*P*-value<5x10^-6^ and r^2^<0.001) based on single nucleotide polymorphisms (SNPs) from a meta-analysis across all SNPs available in three data sources (CORtisol NETwork (CORNET) consortium, Shin GWAS and Long GWAS) using p-value based effect size with sample overlap correction with ischemic heart disease (IHD) based on the CARDIoGRAMplusC4D 1000 Genomes-based GWAS (1000 Genomes) with replication based on the UK Biobank using Mendelian randomization (MR) with different methods

| Exposure | Outcome | SNPs | *F-statistic* | Method | Odds | 95% CI |  | *P-value* | IVW |  |  | MR-Egger | |
| --- | --- | --- | --- | --- | --- | --- | --- | --- | --- | --- | --- | --- | --- |
| sources | sources |  |  |  | ratio |  |  |  | Cochran’s  *Q*-statistic | *P*-value |  | Intercept  *P*-value | I^2^ |
| Meta-analysis | 1000 Genomes | 42 | 23.4 | IVW | 1.01 | 0.99 | 1.03 | 0.40 | 49.84 | 0.16 |  |  |  |
|  |  |  |  | WM | 1.00 | 0.98 | 1.03 | 0.82 |  |  |  |  |  |
|  |  |  |  | MR-Egger | 1.01 | 0.98 | 1.04 | 0.42 |  |  |  | 0.71 | 93.4% |
|  |  |  |  | MR-PRESSO | 1.01 | 0.99 | 1.03 | 0.40 |  |  |  |  |  |
|  | UK Biobank | 40 | 23.5 | IVW | 0.99 | 0.97 | 1.01 | 0.27 | 35.54 | 0.63 |  |  |  |
|  |  |  |  | WM | 0.99 | 0.97 | 1.02 | 0.53 |  |  |  |  |  |
|  |  |  |  | MR-Egger | 0.99 | 0.96 | 1.01 | 0.30 |  |  |  | 0.67 | 93.4% |
|  |  |  |  | MR-PRESSO | 0.99 | 0.97 | 1.01 | 0.25 |  |  |  |  |  |

Abbreviations: CI, confidence interval; IVW, inverse variance weighting; MR, Mendelian randomization, SNP, single nucleotide polymorphism; WM, weighted median.

Table S3. Association of genetically predicted cortisol (*P*-value<5x10^-6^ and r^2^<0.001) based on single nucleotide polymorphisms (SNPs) from a meta-analysis across all SNPs available in three data sources (CORtisol NETwork (CORNET) consortium, Shin GWAS and Long GWAS) using p-value based effect size with sample overlap correction with ischemic stroke based on the MEGASTROKE using Mendelian randomization (MR) with different methods

| Exposure | Outcome | SNPs | *F*-statistic | Method | Odds | 95% CI |  | *P*-value | IVW | |  | MR-Egger | |
| --- | --- | --- | --- | --- | --- | --- | --- | --- | --- | --- | --- | --- | --- |
| sources | sources |  |  |  | ratio |  |  |  | Cochran’s  *Q*-statistic | *P*-value |  | Intercept  *P*-value | I^2^ |
| Meta-analysis | MEGASTROKE | 42 | 23.4 | IVW | 1.00 | 0.98 | 1.02 | 0.84 | 38.45 | 0.58 |  |  |  |
|  |  |  |  | WM | 1.00 | 0.98 | 1.03 | 0.84 |  |  |  |  |  |
|  |  |  |  | MR-Egger | 1.02 | 0.98 | 1.05 | 0.33 |  |  |  | 0.28 | 93.4% |
|  |  |  |  | MR-PRESSO | 1.00 | 0.98 | 1.02 | 0.83 |  |  |  |  |  |

Abbreviations: CI, confidence interval; IVW, inverse variance weighting; MR, Mendelian randomization, SNP, single nucleotide polymorphism; WM, weighted median.

Table S4. Association of genetically predicted cortisol (*P*-value<5x10^-6^ and r^2^<0.001) based on single nucleotide polymorphisms (SNPs) from a meta-analysis across all SNPs available in three data sources (CORtisol NETwork (CORNET) consortium, Shin GWAS and Long GWAS) using p-value based effect size with sample overlap correction with type 2 diabetes (T2DM) based on the DIAbetes Meta-ANalysis of Trans-Ethnic association studies (DIAMANTE) with checking based on the UK Biobank using Mendelian randomization (MR) with different methods

| Exposure | Outcome | SNPs | *F*-statistic | Method | Odds | 95% CI |  | *P*-value | IVW | |  | MR-Egger | |
| --- | --- | --- | --- | --- | --- | --- | --- | --- | --- | --- | --- | --- | --- |
| sources | sources |  |  |  | ratio |  |  |  | Cochran’s  *Q*-statistic | *P*-value |  | Intercept  *P*-value | I^2^ |
| Meta-analysis | DIAMANTE^a^ | 38 | 23.5 | IVW | 0.99 | 0.98 | 1.01 | 0.52 | 77.87 | 0.0001 |  |  |  |
|  |  |  |  | WM | 0.99 | 0.97 | 1.01 | 0.17 |  |  |  |  |  |
|  |  |  |  | MR-Egger | 0.99 | 0.96 | 1.02 | 0.45 |  |  |  | 0.65 | 93.3% |
|  |  |  |  | MR-PRESSO ^a^ | 0.99 | 0.97 | 1.00 | 0.09 |  |  |  |  |  |
|  | UK Biobank | 40 | 23.5 | IVW | 1.00 | 0.98 | 1.03 | 0.77 | 43.85 | 0.27 |  |  |  |
|  |  |  |  | WM | 1.00 | 0.97 | 1.03 | 0.89 |  |  |  |  |  |
|  |  |  |  | MR-Egger | 0.98 | 0.95 | 1.01 | 0.27 |  |  |  | 0.09 | 93.4% |
|  |  |  |  | MR-PRESSO | 1.00 | 0.98 | 1.03 | 0.77 |  |  |  |  |  |

Abbreviations: CI, confidence interval; IVW, inverse variance weighting; MR, Mendelian randomization, SNP, single nucleotide polymorphism; WM, weighted median.

^a^MR-PRESSO estimate was obtained by excluding 1 outlier (*rs4326410*).
